# Effects of breath-hold reproducibility on proton and photon lung cancer stereotactic body radiotherapy

**DOI:** 10.1016/j.phro.2026.100926

**Published:** 2026-02-17

**Authors:** Nils Olovsson, Kenneth Wikström, Anna Flejmer, Alexandru Dasu

**Affiliations:** a Department of Immunology, Genetics and Pathology, Uppsala University, Uppsala, Sweden; b The Skandion Clinic, Uppsala, Sweden; c Department of Medical Physics, Uppsala University Hospital, Uppsala, Sweden; d Department of Oncology, Uppsala University Hospital, Uppsala, Sweden

**Keywords:** Lung cancer, Breath-hold, SBRT, Proton therapy, Robust optimization, Probabilistic evaluation

## Abstract

**Background and purpose:**

Breath-hold can mitigate respiratory motion in lung cancer radiotherapy. Reduced motion could be especially beneficial for proton therapy which is more sensitive to geometrical perturbations than photon therapy. However, failure to reproduce the breath-hold tumor position could lessen this advantage. In this study, effects of reproducibility were investigated for 3D and 4D robust optimized proton and photon therapy.

**Materials and methods:**

Fourteen patients with early stage lung cancer, imaged with a single breath-hold computed tomography, were included. Reproducibility variations were included in the treatment planning and simulated with image deformations. Further, additional larger variations were included in the evaluation.

Three photon and three proton therapy treatment plans were robustly optimized for the same intended dose and compared using probabilistic evaluation. One 3D method only accounted for patient shifts. A second 3D method accounted for breath-hold variations using larger patient shifts. The final 4D method used additional planning images.

**Results:**

Protons resulted in reduced dose to organs of interest but with higher spread in target dose compared with photons. Mean heart dose was reduced, 90% probability of 6.7 Gy with photons compared with 0.3 Gy for protons for 4D planning. Decreased reproducibility affected tumor dose ranges. However, the 90% probability of median tumor dose remained stable.

**Conclusions:**

Reduction in dose to organs of interest was demonstrated with proton therapy. Using 4D compared with 3D optimization did not result in more robust plans. Larger breath-hold variability than anticipated during planning had a minor effect on the target dose.

## Highlights

• Proton and photon stereotactic body radiotherapy in breath-hold was investigated.
• Two different methods to account for variations in breath-holds were compared.
• Twice as large breath-hold variations than in treatment planning were simulated.
• Robust treatment planning successfully accounted for variations in breath-hold.
• Proton therapy reduced the mean heart dose to near 0 Gy.

## Graphical abstract

**Figure:**
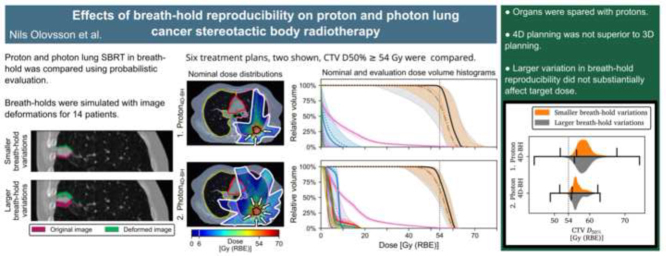

## 1 Introduction

Breath-hold (BH) gated radiotherapy was introduced to manage respiratory motion, reduce lung dose [1], and ensure target coverage [2], [3], [4]. Treatment in BH requires compliance with guidelines recommending patients to recover and retain the BH position for at least 20 seconds [5].

However, variations in the reproduced tumor position between BHs risk worsening target coverage. These variations can be caused by strain, lack of feedback, or a low correlation of the external surrogate signal used for visual guidance to the internal motion [5]. Further, discrepancies as investigated using consecutive computed tomographies (CT) in BH (BHCT) [6], cone-beam computed tomography [7], [8], or both [9], cine-CT [10], or fiducial markers [11] have been reported.

Stereotactic body radiotherapy (SBRT) [12] is the standard of care for inoperable patients with early stage lung cancer and has shown superior outcome compared with conventionally fractionated treatments [13]. Proton therapy is emerging as an alternative to photon therapy [14], [15] with a possibility of reducing dose to organs of interest (OOI). The cost of this tissue sparing is a potentially larger variation in target coverage [16] which stems from the sensitivity of protons to geometric perturbations, changes in anatomy [14], and conversion of CT to stopping power ratio (SPR) for proton dose computations [17].

Clinical target volume (CTV) coverage has been demonstrated for robust optimized photon SBRT [18], [19], [20] and for BH proton therapy in a preliminary, single case study [16]. However, that study was limited as different robust planning approaches and discrepancies in the ability to reproduce BH positions were not investigated.

In this study proton and photon SBRT in BH with two different approaches to account for variations in BH positioning in robust treatment planning were compared. As such, three planning approaches with different degrees of incorporating variations in patient positioning and BH were explored. Further, effects on dose of larger variations in BH reproducibility than assumed during planning were investigated. The plans were compared using simulations with deformed BHCTs in a probabilistic evaluation. Dose distributions for three treatment planning approaches were analyzed for 14 patients using two different sets of values for BH reproducibility.

## 2 Materials and methods

### 2.1 Patient image data and simulation parameters

Image data from 14 patients that were candidates for lung SBRT was used [21], approved by the Swedish Ethical Review Authority, number 2020-00816. The BHCTs were acquired at comfortable, near-deep, inspiration position.

Systematic and fraction-wise standard deviations in patient setup (PS) positioning [22], denoted $\Sigma_{\text{PS}}$ and $\sigma_{\text{PS}}$, and corresponding values for systematic and per-BH tumor position variations [6], $\Sigma_{\text{BH-A}}$ and $\sigma_{\text{BH-A}}$, were retrieved from the literature and used for planning and evaluation, Table 1. A second set, $\Sigma_{\text{BH-B}}$ and $\sigma_{\text{BH-B}}$, was further used only for evaluation [9]. These were labeled A and B where the latter represented a larger variation in BH tumor position reproducibility. A composite value for the standard deviation in SPR for thoracic treatments, $\Sigma_{\text{SPR}}$, was used for protons [17].

**Table 1:** Standard deviations for the parameters used in the robust planning and evaluation. Σ refers to systematic variations that persist during the whole treatment and σ to random variations that are different at each fraction or breath-hold. Breath-hold tumor position variations were modeled using two sets of values, A and B. The positional standard deviations are given in the left–right (LR), anterior–posterior (AP), and craniocaudal (CC) directions.

	LR	AP	CC
Patient setup positioning [mm] [22]			
$\Sigma_{\text{PS}}$	1.1	1.5	1.4
$\sigma_{\text{PS}}$	1.4	1.7	1.7

Breath-hold tumor position [mm] [6]			
$\Sigma_{\text{BH-A}}$	1.3	1.2	1.1
$\sigma_{\text{BH-A}}$	0.9	1.0	1.0

Breath-hold tumor position [mm] [9]			
$\Sigma_{\text{BH-B}}$	1.0	1.2	2.2
$\sigma_{\text{BH-B}}$	1.1	1.6	2.7

Stopping power ratio [%] [17]			
$\Sigma_{\text{SPR}}$		3.8	

### 2.2 Breath-hold image deformation

**Fig. 1:**
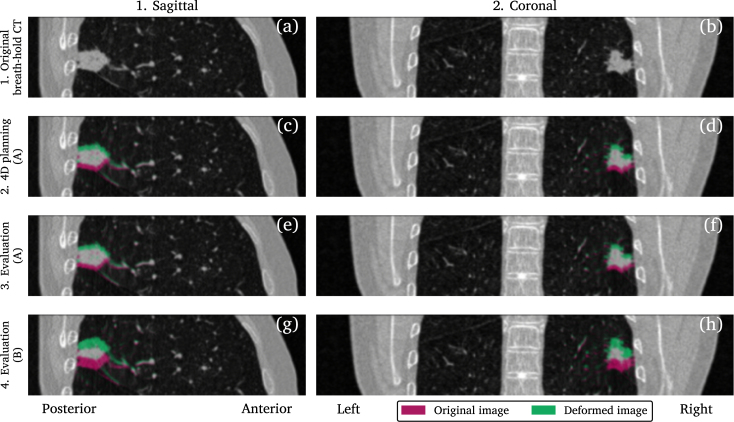
Original and deformed breath-hold planning CT illustrated for patient 4. The original image is shown in (a) and (b) while (c) to (h) show the images with the largest tumor displacement in the craniocaudal direction used to create the respective image set. Two sets of values were used to model the variations in breath-hold tumor position, A and B. Since the maximum craniocaudal displacements used to generate the images shown in (c) to (f) were very similar these resulting images were also similar.

The BHCTs were deformed using Laplacian displacement vector fields [16] to simulate variations in BH tumor positions. Four image sets, including the original BHCT, were created for each patient. The original BHCT, Figs. 1 a and b, was used for robust 3D treatment planning.

The first deformed image set was used for robust 4D treatment planning, created using tumor shifts according to the 90% confidence interval [23] of the combined maximum systematic and per-BH standard deviations, max($\Sigma_{\text{BH-A}}$) and max($\sigma_{\text{BH-A}}$) in Table 1 according to a previously described method [16]. The shifts were sampled isotropically at positions along the axes which resulted in seven deformed images, Figs. 1 c and d, including one non-deformed. These simulated repeated BHCT images that would be acquired for treatment planning.

The second set of deformed images, evaluation image set A, was used for probabilistic evaluation and were created from the same set of standard deviations as the first image set but sampled differently. Tumor shifts in this image set were divided into systematic and per-BH shifts that were combined to a shift used to deform the image. These shifts were calculated using $\Sigma_{\text{BH-A}}$ and $\sigma_{\text{BH-A}}$ in Table 1 [6], sampled, and probability weighted according to a previously described method [16]. When combining discrete systematic and per-BH tumor shifts it resulted in 105 deformed images, as described in [16], Figs. 1 e and f.

The third and final deformed image set, evaluation image set B, was based on overall larger variations in reproducibility, $\Sigma_{\text{BH-B}}$ and $\sigma_{\text{BH-B}}$ in Table 1 [9]. These resulted in larger tumor shifts, sampled according to the same pattern as the second image set for a total of 105 deformed images, Figs. 1 g and h. These were used for a second probabilistic evaluation aiming to investigate plan robustness to discrepancies in reproducibility.

To prevent impossible deformations, tumor shifts were constrained by the lung wall. Each displacement deformed the image while its inverse was used to allow dose accumulation on the reference geometry.

### 2.3 Treatment planning and dose computations

Treatment planning and dose computations were performed with a research version (11B) of RayStation (RaySearch Laboratories AB, Stockholm, Sweden). Photon dose computations were performed using collapsed cone (v5.6) and proton doses using Monte Carlo (v5.3) algorithm with constant relative biological effectiveness (RBE) of 1.1 [24], Monte Carlo uncertainty of 2%, and grid cell size of 3mm×3mm×3mm.

**Fig. 2:**
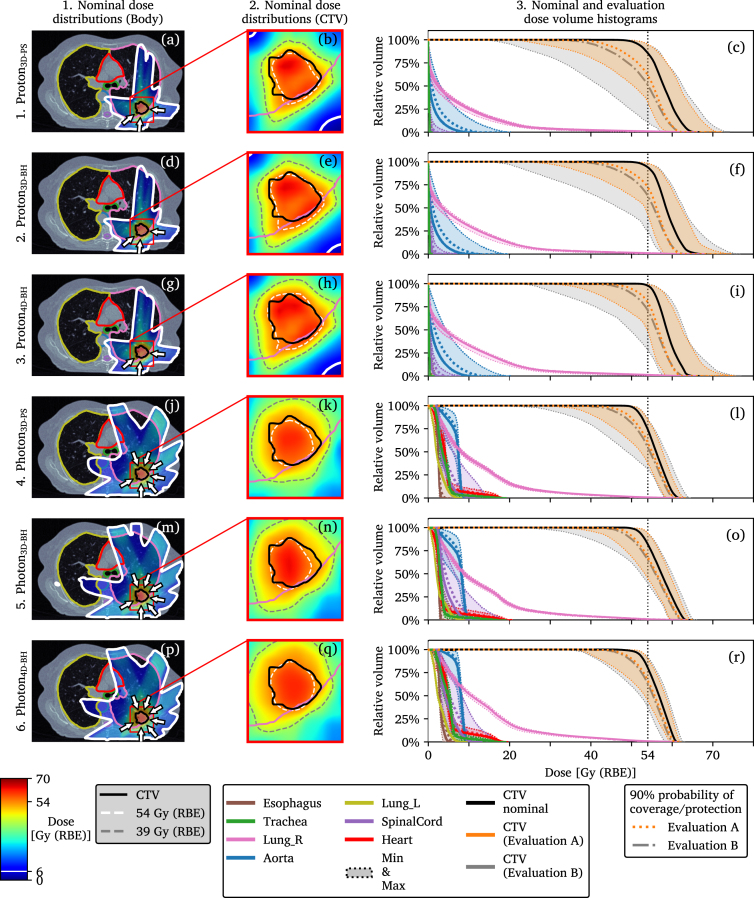
Dose distributions and dose volume histograms (DVH) shown per plan for patient 4. The nominal dose distributions are shown for the entire transversal image plane that intersects the center of the tumor in (a), (d), (g), (j), (m), and (p) and as a zoomed in view of the same image plane in (b), (e), (h), (k), (n), and (q). The solid DVH curves in (c), (f), (i), (l), (o), and (r) indicate the nominal values and the shaded regions the range of DVH values during the two evaluations, A and B. A line indicating a 90% probability, $p_{90\text{\%}}$, of being to the right of that curve is drawn for both evaluations for the clinical target volume (CTV). An analogous line indicating a 90% probability of protecting an organ of interest (OOI) against higher doses is indicated for only the evaluations performed with the evaluation image set A. This same type of illustration for all fourteen patients can be found in Supplementary Figures S1–S14.

All plans prioritized a sharp dose fall-off outside of the volume occupied by the CTV and high dose to chest wall was penalized. Dose to OOIs was mostly minimized by the beam angle arrangement. The photon plans used six or seven beam directions, illustrated in Fig. 2, optimized for static field openings. All proton plans used three beam directions and were planned using an IBA pencil beam scanning beam model. However, whenever one individual beam contained more than ten energy layers it was split into two sub-beams each containing one half of the successive energy layers [16]. The purpose of this was to get sub-beams that could be delivered during a single BH [5].

### 2.4 Robust optimization, evaluation, and normalization

All plans were robustly optimized [25] and had the same intention of delivering a dose in three fractions such that CTV $D_{50\text{\%}}$ ≥ 54 Gy [26], [27]. Robust optimization that used only shifted patient positions, denoted as 3D, or in combination with additional planning images, denoted as 4D, was performed. The perturbations used for robust optimization scenarios varied across the three treatment planning strategies and are summarized in Table 2. A total of six plans per patient were created and evaluated. All proton plans used three SPR perturbations of −4.7%, 0% and 4.7%, calculated using a previously described method [16].

The first strategy employed 3D robust optimization with patient shifts of 5.7 mm to account only for variations in patient positioning, sampled at 15 positions [16]. Potential BH reproducibility variations were not accounted for. These plans were denoted ${\text{proton}}_{\text{3D-PS}}$ and ${\text{photon}}_{\text{3D-PS}}$ and optimized 15 scenarios for ${\text{photon}}_{\text{3D-PS}}$ and 3×15=45 scenarios for ${\text{proton}}_{\text{3D-PS}}$.

The second strategy employed 3D robust optimization with larger patient shifts of 7 mm to simultaneously accommodate both variations in patient positioning and BH reproducibility [16]. These plans were denoted ${\text{proton}}_{\text{3D-BH}}$ and ${\text{photon}}_{\text{3D-BH}}$ and optimized 15 scenarios for ${\text{photon}}_{\text{3D-BH}}$ and 3×15=45 scenarios for ${\text{proton}}_{\text{3D-BH}}$.

The third strategy employed 4D robust optimization with patient shifts of 5.7 mm to accommodate only variations in patient positioning. The variation in BH reproducibility was considered using the 7 deformed planning BHCT images. These plans were denoted ${\text{proton}}_{\text{4D-BH}}$ and ${\text{photon}}_{\text{4D-BH}}$ and optimized 15×7=105 scenarios for ${\text{photon}}_{\text{4D-BH}}$ and 3×15×7=315 scenarios for ${\text{proton}}_{\text{4D-BH}}$.

A robust evaluation was performed by applying all perturbations used for robust optimization with the dose being recomputed for each image used for planning, i.e. one image for the 3D optimized plans and seven images for the 4D optimized plans. With the intention to harmonize CTV coverage across patients all plans were normalized such that the robust scenario with lowest CTV $D_{50\text{\%}}$ was equal to 54 Gy [16]. As such, all treatment plans were guaranteed to fulfill the robust CTV prescription.

**Table 2:** Perturbations used for the robust treatment planning. The plans labeled BH all incorporated variations in patient positioning as well as breath-hold reproducibility while the two plans labeled PS only accommodated for variations in patient positioning. The plans labeled 4D used multiple images in the robust optimization while the ones labeled 3D used only the original breath-hold CT. Variations in stopping power ratio (SPR) conversion was only considered for proton therapy.

Plan	Isotropic	Isotropic tumor shift in	SPR [%]
	patient shift [mm]	deformed planning CTs [mm]	
${\text{proton}}_{\text{3D-PS}}$	5.7	0.0	4.7
${\text{proton}}_{\text{3D-BH}}$	7.0	0.0	4.7
${\text{proton}}_{\text{4D-BH}}$	5.7	4.1	4.7

${\text{photon}}_{\text{3D-PS}}$	5.7	0.0	0.0
${\text{photon}}_{\text{3D-BH}}$	7.0	0.0	0.0
${\text{photon}}_{\text{4D-BH}}$	5.7	4.1	0.0

### 2.5 Probabilistic evaluation and analysis

Probabilistic evaluations were performed where precomputed dose distributions corresponding to combinations of discrete variations were sampled according to their probability [16]. Variations were divided into systematic SPR perturbations, patient position, and BH position as well as fraction-wise variations in patient position and per-BH variations. Using this evaluation schema, 10 000 scenario dose distributions per each of the 14 patients, the six treatment plans, and the two evaluation image sets, A and B, created as described in Section 2.2, were simulated. This resulted in 10000×14×6×2=1680000 simulated treatments.

Results from all patients were grouped according to planning method and evaluation image set, resulting in 140000 samples per group. Dose-volume values and dose volume histograms (DVH) were computed. Homogeneity index (HI) defined by

(1) $$HI=\frac{D_{2\text{\%}}-D_{98\text{\%}}}{D_{50\text{\%}}}$$

was reported for CTV [28].

Probability to reach at least a dose-volume value with 90% certainty for CTV was defined by $p_{90\text{\%}}$ and calculated as the 10th percentile. For CTV HI and OOIs, $p_{90\text{\%}}$ defined a 90% certainty of avoiding a higher value or dose, respectively, calculated as the 90th percentile. Planning methods were compared using the average of the nominal, without any simulated perturbations as seen during treatment planning, dose-volume values and using $p_{90\text{\%}}$. Effects of different variations in BH tumor position reproducibility were compared using $p_{90\text{\%}}$.

## 3 Results

### 3.1 Comparison of proton and photon planning strategies

Generally, proton plans resulted in higher CTV $D_{50\text{\%}}$ compared with photon plans. Averaged across the 14 patients the proton plans, denoted by 3D-PS, 3D-BH and 4D-BH, had arithmetic mean nominal CTV $D_{50\text{\%}}$ values of 57.9, 58.4 and 59.0 Gy while the corresponding values for photons were 55.8, 56.6 and 56.9 Gy. The CTV and OOI results are summarized in Table 3. Metrics for near-minimum and near-maximum doses for the CTV can be found in Supplementary Table S1.

The $p_{90\text{\%}}$ of CTV $D_{50\text{\%}}$ during evaluation with image set A were lower than the nominal values with 55.0, 55.8 and 56.3 Gy for proton plans and 54.0, 54.5 and 55.0 Gy for photon plans.

**Table 3:** Results for all patients and all simulated treatments. The nominal plan and evaluation results are reported for the clinical target volume (CTV) and organs of interest as dose covering x% of the volume ($D_{x\text{\%}}$), mean dose ($D_{\text{mean}}$), or voxel-wise maximum dose ($D_{0\text{\%}}$) of the respective volume. The upper, first, part of the table is reporting results from the three proton therapy plans while the lower, second, half is reporting results for the three photon therapy plans. For the CTV the homogeneity index (HI) is also reported. Results for near-minimum and near maximum CTV dose can be found in the Supplementary Table S1. Treatment plans were created using the breath-hold (BH) reproducibility values A and evaluated using the image sets created using both values A and B. ^†^Planning and evaluation. *Evaluation only.

		Nom.	Evaluation	Nom.	Evaluation	Nom.	Evaluation
Number of samples		14	140000	14	140000	14	140000
Summary statistics		Avg. (SD)	Med. (IQR) [Min, Max] ($p_{90\text{\%}}$)	Avg. (SD)	Med. (IQR) [Min, Max] ($p_{90\text{\%}}$)	Avg. (SD)	Med. (IQR) [Min, Max] ($p_{90\text{\%}}$)
(1) Proton plans		${\text{proton}}_{\text{3D-PS}}$		${\text{proton}}_{\text{3D-BH}}$		${\text{proton}}_{\text{4D-BH}}$	

A^†^	CTV $D_{50\text{\%}}$ [Gy]	57.9 (1.1)	56.9 (2.1) [46.5, 62.0] (55.0)	58.4 (1.1)	57.8 (1.8) [49.5, 63.7] (55.8)	59.0 (1.6)	58.3 (2.3) [51.7, 67.7] (56.3)
	CTV HI	0.16 (0.05)	0.22 (0.10) [0.07, 0.76] (0.33)	0.15 (0.05)	0.20 (0.10) [0.06, 0.74] (0.30)	0.14 (0.05)	0.17 (0.09) [0.05, 0.57] (0.27)

B*	CTV $D_{50\text{\%}}$ [Gy]	*	56.4 (2.6) [34.5, 64.7] (53.7)	*	57.3 (2.4) [40.4, 68.6] (54.9)	*	58.0 (2.6) [44.3, 74.2] (55.7)
	CTV HI	*	0.25 (0.13) [0.07, 1.15] (0.40)	*	0.22 (0.12) [0.06, 1.16] (0.36)	*	0.20 (0.11) [0.05, 0.85] (0.32)

A^†^	Lung $V_{5\text{Gy}}$ [cm^3^]	303 (118)	310 (210) [54, 514] (459)	331 (121)	341 (211) [61, 538] (477)	357 (124)	365 (204) [72, 565] (511)
	Lung $V_{20\text{Gy}}$ [cm^3^]	91 (44)	84 (53) [18, 211] (177)	105 (46)	99 (60) [21, 230] (185)	127 (53)	125 (65) [35, 261] (221)
	Heart $D_{0\text{\%}}$ [Gy]	3.1 (6.8)	0.1 (1.3) [0.0, 26.6] (18.2)	3.1 (6.6)	0.1 (1.8) [0.0, 26.3] (18.0)	3.4 (7.1)	0.1 (2.6) [0.0, 27.4] (19.5)
	Heart $D_{\text{mean}}$ [Gy]	0.0 (0.1)	0.0 (0.0) [0.0, 0.5] (0.2)	0.0 (0.1)	0.0 (0.0) [0.0, 0.5] (0.3)	0.1 (0.1)	0.0 (0.0) [0.0, 0.6] (0.3)
	Esophagus $D_{0\text{\%}}$ [Gy]	1.5 (2.2)	0.1 (2.9) [0.0, 13.1] (4.9)	1.6 (2.4)	0.1 (3.4) [0.0, 12.9] (5.3)	1.8 (2.7)	0.1 (3.0) [0.0, 14.0] (6.3)
	Aorta $D_{0\text{\%}}$ [Gy]	5.9 (3.7)	5.7 (6.2) [0.0, 21.3] (11.7)	6.0 (3.8)	5.7 (6.4) [0.0, 21.4] (12.0)	6.2 (4.0)	5.9 (6.5) [0.0, 22.9] (12.9)
	Spinal cord $D_{0\text{\%}}$ [Gy]	1.4 (2.0)	0.2 (2.2) [0.0, 13.3] (5.8)	1.7 (2.0)	0.4 (2.8) [0.0, 15.9] (5.9)	2.0 (2.5)	0.4 (3.5) [0.0, 14.5] (7.0)

B*	Lung $V_{5\text{Gy}}$ [cm^3^]	*	310 (209) [54, 514] (459)	*	341 (211) [60, 538] (477)	*	365 (204) [71, 565] (512)
	Lung $V_{20\text{Gy}}$ [cm^3^]	*	83 (53) [16, 211] (176)	*	99 (60) [21, 232] (185)	*	125 (65) [35, 265] (221)
	Heart $D_{0\text{\%}}$ [Gy]	*	0.1 (1.4) [0.0, 27.4] (17.9)	*	0.1 (1.8) [0.0, 27.6] (18.2)	*	0.1 (2.6) [0.0, 29.8] (19.5)
	Heart $D_{\text{mean}}$ [Gy]	*	0.0 (0.0) [0.0, 0.5] (0.2)	*	0.0 (0.0) [0.0, 0.5] (0.3)	*	0.0 (0.0) [0.0, 0.6] (0.3)
	Esophagus $D_{0\text{\%}}$ [Gy]	*	0.1 (2.9) [0.0, 13.2] (5.4)	*	0.1 (3.4) [0.0, 12.7] (5.5)	*	0.1 (3.0) [0.0, 14.2] (6.3)
	Aorta $D_{0\text{\%}}$ [Gy]	*	5.9 (6.2) [0.0, 21.9] (11.9)	*	5.9 (6.4) [0.0, 21.9] (12.1)	*	6.1 (6.5) [0.0, 23.2] (13.0)
	Spinal cord $D_{0\text{\%}}$ [Gy]	*	0.2 (2.2) [0.0, 13.3] (5.8)	*	0.4 (2.7) [0.0, 15.7] (5.9)	*	0.4 (3.5) [0.0, 14.3] (7.0)

(2) Photon plans		${\text{photon}}_{\text{3D-PS}}$		${\text{photon}}_{\text{3D-BH}}$		${\text{photon}}_{\text{4D-BH}}$	

A^†^	CTV $D_{50\text{\%}}$ [Gy]	55.8 (1.4)	54.9 (1.9) [44.6, 61.2] (54.0)	56.6 (1.6)	55.6 (2.3) [49.7, 62.7] (54.5)	56.9 (1.4)	56.3 (1.7) [51.5, 62.3] (55.0)
	CTV HI	0.17 (0.03)	0.20 (0.06) [0.11, 0.62] (0.27)	0.17 (0.04)	0.19 (0.06) [0.08, 0.54] (0.25)	0.15 (0.02)	0.17 (0.04) [0.10, 0.39] (0.21)

B*	CTV $D_{50\text{\%}}$ [Gy]	*	54.7 (2.0) [38.1, 61.5] (53.5)	*	55.4 (2.5) [44.5, 63.0] (54.2)	*	56.2 (1.8) [48.8, 62.9] (54.8)
	CTV HI	*	0.21 (0.07) [0.10, 0.75] (0.30)	*	0.20 (0.07) [0.08, 0.67] (0.28)	*	0.17 (0.05) [0.08, 0.46] (0.22)

A^†^	Lung $V_{5\text{Gy}}$ [cm^3^]	474 (135)	453 (256) [256, 709] (664)	497 (139)	477 (278) [268, 734] (672)	538 (141)	569 (266) [265, 772] (703)
	Lung $V_{20\text{Gy}}$ [cm^3^]	112 (55)	93 (73) [39, 260] (213)	129 (57)	106 (70) [56, 279] (235)	158 (71)	128 (94) [73, 360] (283)
	Heart $D_{0\text{\%}}$ [Gy]	13.9 (4.6)	14.8 (9.1) [8.3, 22.1] (19.9)	14.6 (4.7)	16.1 (9.0) [8.7, 23.0] (20.5)	14.4 (4.9)	15.5 (8.9) [8.9, 24.3] (22.1)
	Heart $D_{\text{mean}}$ [Gy]	4.1 (1.1)	4.3 (2.3) [2.4, 6.4] (5.5)	4.2 (1.2)	3.8 (2.8) [2.6, 7.4] (6.1)	4.7 (1.4)	5.1 (2.5) [2.5, 7.2] (6.7)
	Esophagus $D_{0\text{\%}}$ [Gy]	8.3 (1.8)	9.0 (2.2) [2.9, 15.3] (10.0)	8.5 (1.9)	8.8 (2.0) [2.9, 14.1] (10.3)	8.9 (1.4)	8.9 (1.7) [4.0, 17.5] (10.3)
	Aorta $D_{0\text{\%}}$ [Gy]	11.5 (2.2)	11.3 (3.4) [7.7, 20.9] (14.5)	11.7 (2.3)	11.2 (3.4) [7.4, 21.9] (14.9)	12.4 (2.7)	12.6 (4.8) [8.1, 21.2] (16.1)
	Spinal cord $D_{0\text{\%}}$ [Gy]	8.9 (2.6)	9.6 (2.2) [1.8, 17.2] (11.6)	9.3 (2.8)	9.9 (1.6) [2.0, 19.9] (11.9)	9.5 (2.9)	10.4 (2.0) [2.2, 19.3] (12.4)

B*	Lung $V_{5\text{Gy}}$ [cm^3^]	*	452 (256) [256, 709] (664)	*	476 (278) [268, 734] (672)	*	569 (266) [265, 772] (703)
	Lung $V_{20\text{Gy}}$ [cm^3^]	*	93 (73) [39, 259] (213)	*	106 (70) [56, 279] (235)	*	128 (94) [73, 360] (283)
	Heart $D_{0\text{\%}}$ [Gy]	*	14.8 (9.1) [8.3, 22.1] (19.9)	*	16.1 (9.0) [8.6, 23.1] (20.5)	*	15.5 (8.9) [8.8, 24.4] (22.1)
	Heart $D_{\text{mean}}$ [Gy]	*	4.3 (2.3) [2.4, 6.4] (5.5)	*	3.8 (2.8) [2.6, 7.4] (6.1)	*	5.1 (2.5) [2.5, 7.2] (6.7)
	Esophagus $D_{0\text{\%}}$ [Gy]	*	9.0 (2.2) [2.8, 15.4] (10.0)	*	8.8 (2.0) [2.9, 14.3] (10.3)	*	8.9 (1.7) [4.2, 17.5] (10.3)
	Aorta $D_{0\text{\%}}$ [Gy]	*	11.3 (3.4) [7.6, 20.9] (14.5)	*	11.2 (3.4) [7.3, 21.8] (14.9)	*	12.6 (4.8) [8.0, 21.3] (16.1)
	Spinal cord $D_{0\text{\%}}$ [Gy]	*	9.6 (2.2) [1.8, 17.2] (11.7)	*	9.9 (1.6) [2.0, 19.8] (12.0)	*	10.4 (2.1) [2.2, 19.3] (12.4)

Nominal plan (Nom.) Average, arithmetic mean (Avg.) Standard deviation (SD) Median (Med.) Interquartile range (IQR)

Nominal dose homogeneity in the CTV was better for proton plans compared with photon plans, as indicated by lower average HI values of 0.16, 0.15, and 0.14 compared with 0.17, 0.17, and 0.15, respectively. However, this did not persist in the evaluation where the $p_{90\text{\%}}$ for the HI was higher for proton plans for both evaluation A, 0.33, 0.30, and 0.27 compared with 0.27, 0.25, and 0.21, and B, 0.40, 0.36, and 0.32 compared with 0.30, 0.28, and 0.22, respectively.

Nominal doses to the OOIs were reduced for protons compared with photons. Mean heart dose was reduced to near zero with protons for both the nominal cases and during evaluation.

Voxel-wise maximum doses, $D_{0\text{\%}}$, to esophagus, aorta, and spinal cord were also reduced with protons compared with photons both in nominal cases and for evaluation $p_{90\text{\%}}$. However, the maximum value for aorta $D_{0\text{\%}}$ was actually higher for the ${\text{proton}}_{\text{4D-BH}}$ plan, 22.9 Gy, compared with the photon plans, 20.9 Gy, 21.9 Gy, and 21.2 Gy in evaluation A, illustrating the cost of robust optimization accounting for BH variations. Similarly, maximum values for heart $D_{0\text{\%}}$ in the evaluation were also higher for protons, 26.6 Gy, 26.3 Gy, and 27.4 Gy compared with 22.1 Gy, 23.0 Gy, and 24.3 Gy for photons in evaluation A. Notably, maximum $D_{0\text{\%}}$ values for heart, esophagus, and aorta for protons were several times higher than the very low values indicated by the average nominal value.

Volumes at dose for ipsilateral lung were reduced with protons compared with photons for both $V_{5\text{Gy}}$ and $V_{20\text{Gy}}$.

For nominal cases CTV $D_{50\text{\%}}$ was increased for both protons and photons when going from 3D-PS to 3D-BH and finally 4D-BH robust optimization. The $p_{90\text{\%}}$ of CTV $D_{50\text{\%}}$ also increased in the same manner. The nominal dose distributions for all plans with evaluation DVHs are illustrated for one patient in Fig. 2 and can be found for all patients together with tumor size and location in Supplementary Figures S1–S14.

### 3.2 Effect of larger variation in BH reproducibility on CTV dose between treatment plans

**Fig. 3:**
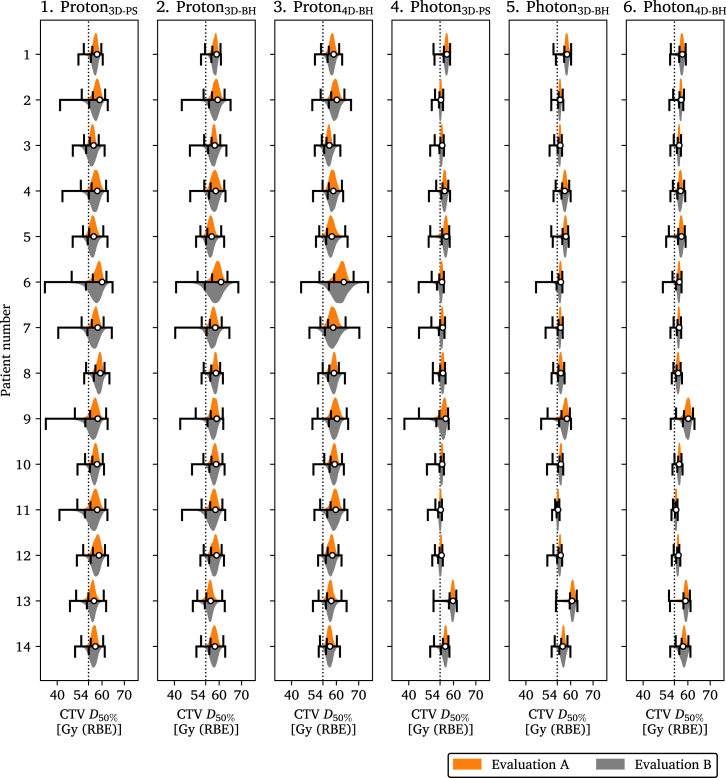
Resulting dose covering 50% ($D_{50\text{\%}}$) of the clinical target volume (CTV) plotted for each patient and treatment plan. The results from the two evaluation image sets, A and B, are shown as orange and gray violin plots. Their $p_{90\text{\%}}$ value is indicated by the shorter black bar on the interval spanned by the smallest and largest values from the probabilistic evaluation, indicated by the longer black bars. The results from the evaluation performed with image set A is presented in the upper halves of the individual plots and the results from the evaluation performed with image set B in the lower. The nominal CTV $D_{50\text{\%}}$ is shown as a white dot. Plan-wise summarized results for all patients can be seen in Supplementary Figure S15 and the same content but plotted to highlight differences between the planning methods can be seen in Supplementary Figure S16.

The results when using evaluation image set B are summarized for all patients and scenarios in Table 3 and presented per patient for CTV $D_{50\text{\%}}$ in Fig. 3. The larger variations in BH reproducibility reduced the $p_{90\text{\%}}$ of the CTV $D_{50\text{\%}}$ for ${\text{proton}}_{\text{3D-PS}}$ by 1.3 Gy, ${\text{proton}}_{\text{3D-BH}}$ by 0.9 Gy, ${\text{proton}}_{\text{4D-BH}}$ by 0.6 Gy, and for ${\text{photon}}_{\text{3D-PS}}$ by 0.5 Gy, ${\text{photon}}_{\text{3D-BH}}$ by 0.3 Gy, and ${\text{photon}}_{\text{4D-BH}}$ by 0.2 Gy. A similar illustration that summarizes resulting CTV $D_{50\text{\%}}$ per plan, and includes results of all patients, can be seen in Supplementary Figure S15 and per plan and patient in Supplementary Figure S16.

Most investigated $p_{90\text{\%}}$ OOI dose-volume values remained unchanged for photons. Proton plans had more changes with the largest being for the esophagus for ${\text{proton}}_{\text{3D-PS}}$ which saw an increase of 0.5 Gy.

## 4 Discussion

In this study we compared proton with photon SBRT in BH using three robustly optimized plans per modality for a total of six plans per patient. Overall results indicate a clear advantage regarding dose to OOIs for protons compared with photons for all patients. Four of the treatment plans accounted for variations in BH reproducibility while two of them only accounted for variations in patient positioning. Further, the effect of variations in the reproduced BH tumor position was investigated using two sets of deformed BHCT evaluation images of which one sampled positions from a different distribution with larger standard deviations than that used during planning. However, the decreased BH reproducibility had only a small impact on target coverage.

The three photon plans displayed more homogeneous and consistent dose to CTV compared with the three proton plans which exhibited more intra-patient variation, see Fig. 2, Fig. 3. When comparing plans of the same modality, the ${\text{proton}}_{\text{4D-BH}}$ and ${\text{photon}}_{\text{4D-BH}}$ plans generally had the highest nominal and evaluation CTV doses followed by ${\text{proton}}_{\text{3D-BH}}$ and ${\text{photon}}_{\text{3D-BH}}$ and finally ${\text{proton}}_{\text{3D-PS}}$ and ${\text{photon}}_{\text{3D-PS}}$, which had the lowest doses. Not accounting for variations in BH reproducibility during optimization gave lower CTV doses compared with the other two plans with the same modality but did not consistently result in minimum CTV $D_{50\text{\%}}$ below 54 Gy. As previously reported, the cost of reduced OOI dose is higher intra-plan variability in dose to CTV for protons compared with photons [16]. It should be noted that due to truncated field-of-views not the full heart and lung volumes were available which affected the results.

Evaluating on image set B the range of the results changed, as seen in the DVHs of Fig. 2, resulting in potentially lower doses to CTV. However, the 90% probability of achieving a certain dose to CTV was not substantially affected by these larger tumor shifts in B. This can be attributed to the very low probability of these events since the standard deviations of the distributions A and B only had differences greater than 1 mm along the craniocaudal direction, Table 1. Spread in resulting CTV doses for all patients, Fig. 3 and supplementary material, might indicate that smaller tumors are more sensitive to treatment variations, see e.g. patients 6, 7, and 9. Although the motion of the tumor and surrounding tissues such as ribs were not treated as independent in the 3D-BH plans, compared with the 4D-BH plans, they still performed well. As such, the increased planning complexity of ${\text{proton}}_{\text{4D-BH}}$ and ${\text{photon}}_{\text{4D-BH}}$ compared with ${\text{proton}}_{\text{3D-BH}}$ and ${\text{photon}}_{\text{3D-BH}}$ did not result in more robust plans, as defined by having a $p_{90\text{\%}}$ CTV $D_{50\text{\%}}$ above the prescription of 54 Gy for both evaluation image sets A and B.

The standard deviations in BH positioning reproducibility used in this study were assessed for patients with locally advanced lung cancer [6], [9] while the simulated treatments were performed for patients with early stage lung cancer. Further, BH reproducibility was modeled at population level while these would be expected to be highly dependent on tumor location. In practice the choice of BH supporting technique would impact reproducibility and intra-fraction monitoring is recommended [5]. A real-time signal of the BH position can be estimated using optical tracking of markers or with surface scanning and can further be used by the patient for visual guidance. Such feedback has been employed in imaging studies [6], [9] and demonstrated for photon SBRT [29] and proton therapy of Hodgkin’s lymphoma [30].

More images for the 4D robust treatment planning could have been generated but this would have expanded the robust optimization problem and might also be less realistic since in the clinical setting only a limited number of repeated BHCTs would be available. In this study seven deformed images were used while typically upwards of three BHCTs are acquired for treatment planning or verification purposes [31], [32]. Sampling tumor shifts only at on-axis positions is a limitation of the 4D planning in this study, however this strategy is typically used for patient positioning offsets in robust optimization [33].

Effects on dose of BH variation on proton and photon therapy has been investigated using repeated imaging for locally advanced disease [31] and SBRT treatments [34] concluding similar benefits of proton therapy regarding dose to OOIs as those observed in the present study. It has further been investigated for photon SBRT using repeated BHCT [35]. The present study did not include any residual motion that might take place during a single BH. Effects of intra-BH motion on photon therapy was investigated using cine-CT [36] suggesting that the impact of any such motion is small.

While including a photon planning method based on treatment margins would have been relevant as a comparison it was outside the scope of the present study. Further, such comparisons have been reported for free-breathing photon treatment planning studies [18], [19], [20]. All proton plans used three beam directions. This choice could have an effect on robustness as fewer directions could worsen target coverage while more could potentially improve it. All treatment plans were conservative with regards to target coverage, using the maximum value of the per-axis reported standard deviations and treating all variations as systematic [16].

The probabilistic evaluation method used in this study was computationally intensive and required the offline storage of a large number of dose distributions corresponding to discrete perturbations to be used for the scenario sampling [16]. Sampling treatment scenarios directly from the continuous distributions and using adequate stopping criteria to estimate probabilities for coverage and avoidance in the form of confidence intervals is an alternative approach that could lessen the computational burden [37], [38]. However, it was demonstrated that the robust evaluation and normalization performed as part of the treatment planning was sufficient and the only thing missed with this approach would be very improbable extreme cases.

In conclusion, proton therapy reduced the dose to OOIs at the cost of a wider spread of final dose in the target compared with photon therapy. Further, accounting for variations in reproducibility of the BH position in treatment planning resulted in higher target dose to accommodate additional geometrical variations. However, none of the plans gave results that were consistently below the intended dose and larger than anticipated variations in BH reproducibility did not substantially affect the 90% probability of achieving a certain dose coverage. In the present study protons lead to favorable SBRT dose distributions for patients with early stage lung cancer, even when variations in e.g. patient and tumor positioning were considered.

## CRediT authorship contribution statement

**Nils Olovsson:** Conceptualization, Methodology, Software, Validation, Formal analysis, Investigation, Data curation, Visualization, Writing – original draft. **Kenneth Wikström:** Supervision, Resources, Writing – review & editing. **Anna Flejmer:** Supervision, Writing – review & editing. **Alexandru Dasu:** Supervision, Funding acquisition, Writing – review & editing.

## Declaration of competing interest

The authors declare the following financial interests/personal relationships which may be considered as potential competing interests: Uppsala University has a research agreement with RaySearch Laboratories AB. No other conflicts of interest have been identified by the authors of the study. The authors declare that they have no known competing financial interests or personal relationships that could have appeared to influence the work reported in this paper.

## Appendix A Supplementary data

MMC S1 Supplementary results table and figures. MMC S1
