# Supplementary material for: Effects of breath-hold reproducibility on proton and photon lung cancer stereotactic body radiotherapy
Source: Phys Imaging Radiat Oncol. 2026 Feb 17;37:100926. doi: 10.1016/j.phro.2026.100926 (PMC12926599; doi:10.1016/j.phro.2026.100926)
Supplement: MMC S1 — Supplementary results table and figures. [file mmc1.pdf]

Effects of breath-hold reproducibility on proton and photon lung cancer stereotactic body radiotherapy - Supplementary material

Nils Olovsson[1,2], Kenneth Wikström[1,3],
Anna Flejmer[1,2,4], Alexandru Dasu[1,2]

[1] Department of Immunology,
Genetics and Pathology, Uppsala University, Uppsala, Sweden
[2] The Skandion Clinic, Uppsala, Sweden
[3] Department of Medical Physics, Uppsala University Hospital, Uppsala, Sweden
[4] Department of Oncology, Uppsala University Hospital, Uppsala, Sweden

Contents

1 CTV dose metrics for each treatment planning method **2**

2 Nominal dose distributions and DVHs for each patient **3**

3 Summarized CTV $D_{50\%}$ per plan for all patients **17**

1 CTV dose metrics for each treatment planning method

Supplementary Table S1: Dosimetric results for the clinical target volume (CTV) for all patients and all simulated treatments. The upper, first, part of the table is reporting results from the three proton therapy plans while the lower, second, half is reporting results for the three photon therapy plans. Treatment plans were created using the breath-hold (BH) reproducibility values A and evaluated using the image sets created using both values A and B. †Planning and evaluation. *Evaluation only.

		Nom.	Evaluation	Nom.	Evaluation	Nom.	Evaluation
	Number of samples	14	140 000	14	140 000	14	140 000
	Summary statistics	Avg. (SD)	Med. (IQR) [Min, Max] ($p_{90\%}$)	Avg. (SD)	Med. (IQR) [Min, Max] ($p_{90\%}$)	Avg. (SD)	Med. (IQR) [Min, Max] ($p_{90\%}$)
**(1) Proton plans**			**proton$_{3D\text{-}PS}$**		**proton$_{3D\text{-}BH}$**		**proton$_{4D\text{-}BH}$**
A†	CTV $D_{99\%}$ [Gy]	52.3 (1.8)	48.4 (4.8) [23.2, 56.8] (42.6)	53.2 (1.7)	50.2 (4.4) [25.4, 57.7] (44.9)	54.4 (2.2)	52.3 (3.9) [28.9, 60.8] (47.8)
	CTV $D_{98\%}$ [Gy]	53.0 (1.6)	49.5 (4.3) [24.6, 57.3] (44.3)	53.9 (1.5)	51.2 (3.8) [27.3, 58.2] (46.5)	54.9 (2.0)	53.0 (3.5) [33.5, 61.4] (49.0)
	CTV $D_{95\%}$ [Gy]	54.1 (1.3)	51.2 (3.5) [28.4, 57.9] (46.9)	54.9 (1.2)	52.6 (3.0) [32.3, 58.6] (48.9)	55.6 (1.9)	54.1 (2.9) [37.8, 62.3] (50.8)
	CTV $D_{2\%}$ [Gy]	62.1 (2.0)	61.7 (3.4) [54.2, 72.7] (58.8)	62.6 (1.9)	62.2 (3.4) [54.6, 72.0] (59.4)	63.0 (2.0)	63.0 (3.8) [54.9, 74.2] (59.9)
	CTV $D_{1\%}$ [Gy]	62.5 (2.2)	62.1 (3.6) [54.4, 74.2] (59.1)	63.1 (2.1)	62.7 (3.6) [54.8, 73.0] (59.7)	63.5 (2.0)	63.5 (3.9) [55.0, 75.0] (60.3)
B*	CTV $D_{99\%}$ [Gy]	*	46.7 (6.0) [15.3, 57.0] (39.3)	*	48.8 (5.5) [17.6, 58.9] (42.1)	*	51.0 (4.7) [19.8, 63.8] (45.6)
	CTV $D_{98\%}$ [Gy]	*	47.9 (5.5) [16.9, 57.4] (41.1)	*	49.8 (4.9) [19.1, 59.6] (43.7)	*	51.8 (4.2) [23.6, 64.4] (47.0)
	CTV $D_{95\%}$ [Gy]	*	49.8 (4.7) [18.7, 58.5] (43.8)	*	51.4 (4.1) [21.5, 60.7] (46.3)	*	53.1 (3.6) [28.2, 66.0] (49.0)
	CTV $D_{2\%}$ [Gy]	*	61.9 (4.1) [51.0, 76.0] (58.4)	*	62.5 (4.0) [48.9, 77.7] (59.1)	*	63.3 (4.2) [50.7, 82.1] (59.8)
	CTV $D_{1\%}$ [Gy]	*	62.3 (4.3) [51.7, 77.6] (58.7)	*	62.9 (4.3) [49.2, 78.6] (59.4)	*	63.8 (4.5) [51.0, 82.9] (60.2)
**(2) Photon plans**			**photon$_{3D\text{-}PS}$**		**photon$_{3D\text{-}BH}$**		**photon$_{4D\text{-}BH}$**
A†	CTV $D_{99\%}$ [Gy]	49.2 (2.2)	47.6 (4.0) [28.2, 54.7] (43.1)	50.1 (2.5)	48.9 (3.7) [30.8, 57.3] (44.9)	50.9 (2.0)	50.2 (3.2) [35.2, 56.5] (46.8)
	CTV $D_{98\%}$ [Gy]	49.9 (2.1)	48.4 (3.7) [28.9, 55.3] (44.3)	50.8 (2.4)	49.6 (3.5) [32.6, 57.9] (45.9)	51.5 (1.8)	50.9 (3.0) [36.7, 57.2] (47.7)
	CTV $D_{95\%}$ [Gy]	50.9 (1.8)	49.6 (3.2) [30.5, 56.1] (46.2)	51.8 (2.2)	50.7 (3.2) [34.4, 58.8] (47.5)	52.5 (1.6)	51.9 (2.5) [39.8, 58.2] (49.1)
	CTV $D_{2\%}$ [Gy]	59.5 (1.6)	58.9 (1.7) [56.5, 65.8] (58.1)	60.2 (1.9)	59.6 (2.2) [56.9, 68.6] (58.3)	60.0 (1.6)	59.5 (2.3) [57.3, 66.4] (58.3)
	CTV $D_{1\%}$ [Gy]	59.7 (1.6)	59.1 (1.7) [56.7, 66.0] (58.2)	60.4 (2.0)	59.8 (2.2) [57.1, 69.2] (58.4)	60.1 (1.6)	59.6 (2.4) [57.4, 67.1] (58.4)
B*	CTV $D_{99\%}$ [Gy]	*	46.8 (4.6) [21.5, 54.9] (41.5)	*	48.3 (4.3) [24.4, 57.5] (43.4)	*	49.8 (3.6) [30.0, 57.2] (45.9)
	CTV $D_{98\%}$ [Gy]	*	47.6 (4.2) [24.7, 55.6] (42.7)	*	49.0 (4.1) [25.5, 58.0] (44.6)	*	50.5 (3.4) [33.8, 57.8] (46.9)
	CTV $D_{95\%}$ [Gy]	*	49.0 (3.7) [25.9, 56.3] (44.8)	*	50.2 (3.7) [28.0, 58.7] (46.4)	*	51.6 (3.0) [36.1, 58.7] (48.4)
	CTV $D_{2\%}$ [Gy]	*	58.8 (1.7) [52.5, 66.0] (57.9)	*	59.6 (2.3) [55.5, 68.8] (58.2)	*	59.4 (2.4) [56.6, 67.9] (58.2)
	CTV $D_{1\%}$ [Gy]	*	59.0 (1.7) [52.8, 66.4] (58.1)	*	59.7 (2.3) [55.8, 69.5] (58.3)	*	59.6 (2.4) [56.8, 68.2] (58.4)

Nominal plan (Nom.) Average, arithmetic mean (Avg.) Standard deviation (SD) Median (Med.) Interquartile range (IQR)

2 Nominal dose distributions and DVHs for each patient

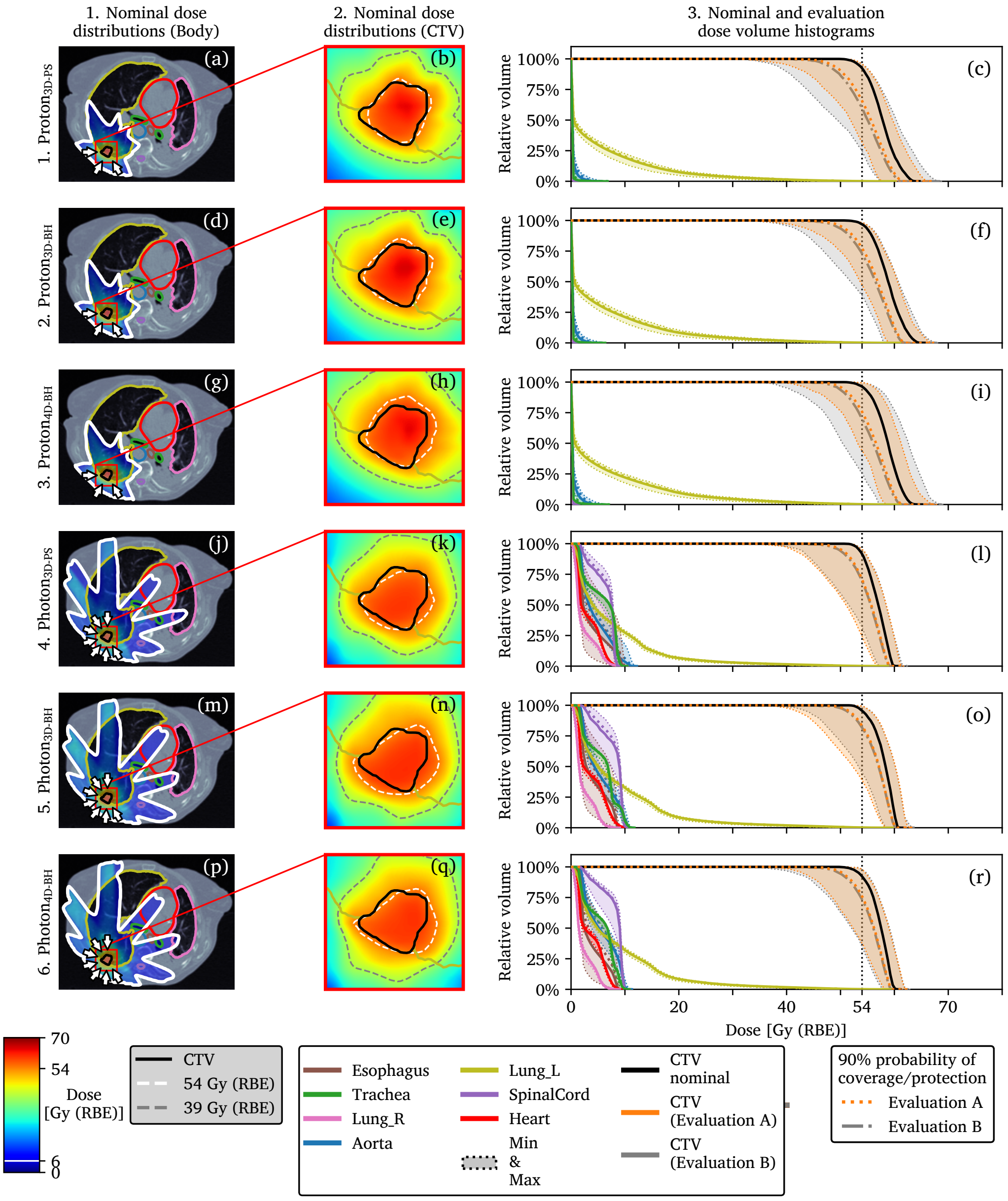

Supplementary Figure S1: Dose distributions and dose volume histograms (DVH) shown per plan for patient 1 with CTV size 4.0 cm^3 and tumor location below the carina in the left lung. The nominal dose distributions are shown for the entire transversal image plane that intersects the center of the tumor in **(a)**, **(d)**, **(g)**, **(j)**, **(m)**, and **(p)** and as a zoomed in view of the same image plane in **(b)**, **(e)**, **(h)**, **(k)**, **(n)**, and **(q)**. The solid DVH curves in **(c)**, **(f)**, **(i)**, **(l)**, **(o)**, and **(r)** indicate the nominal values and the shaded regions the range of DVH values during the two evaluations, A and B. A line indicating a 90% probability, $p_{90\%}$, of being to the right of that curve is drawn for both evaluations for the clinical target volume (CTV). An analogous line indicating a 90% probability of protecting an organ of interest (OOI) against higher doses is indicated for only the evaluations performed with the evaluation image set A.

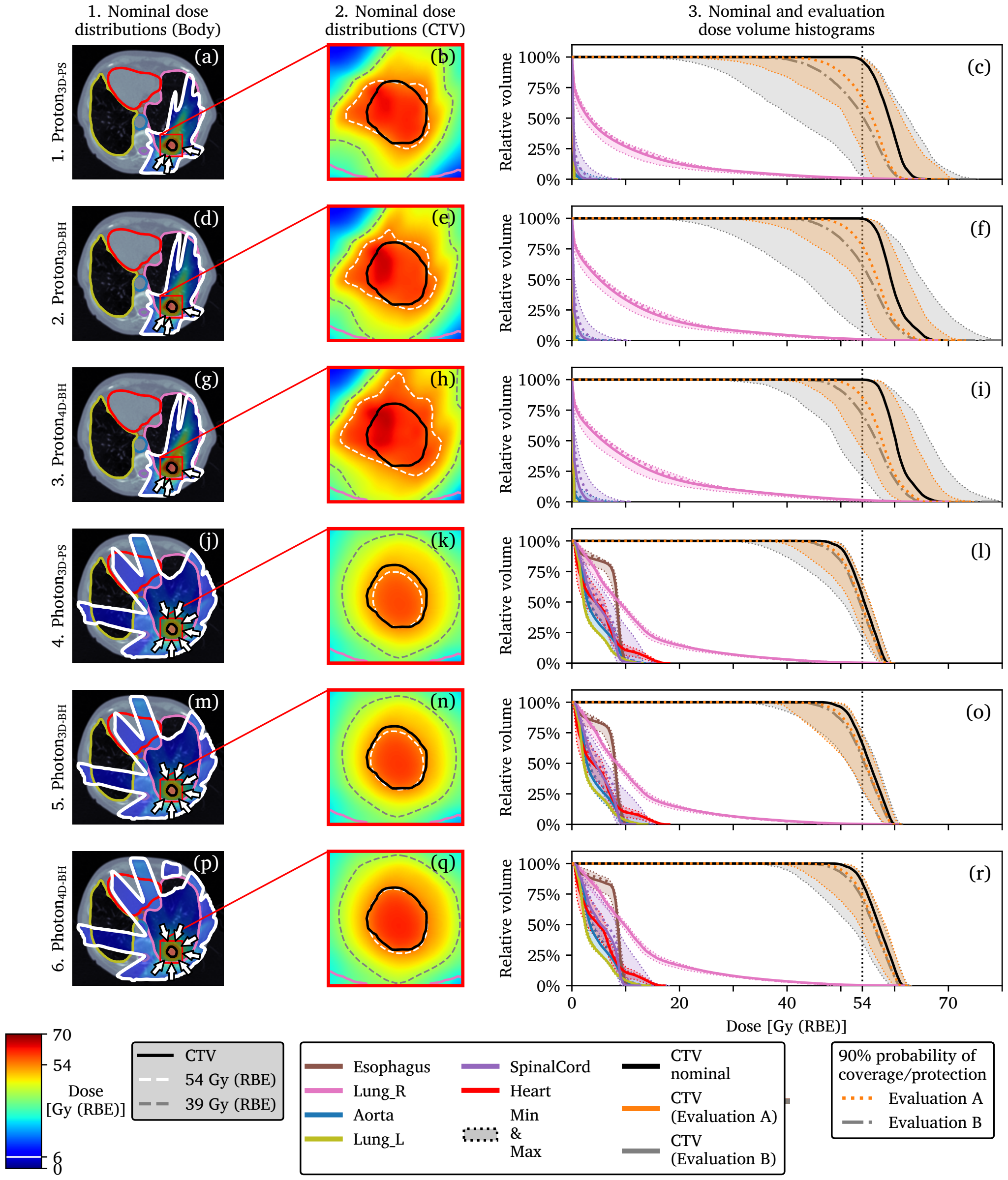

Supplementary Figure S2: Dose distributions and dose volume histograms (DVH) shown per plan for patient 2 with CTV size 3.8 cm^3 and tumor location in the lower lobe in the right lung. The nominal dose distributions are shown for the entire transversal image plane that intersects the center of the tumor in **(a)**, **(d)**, **(g)**, **(j)**, **(m)**, and **(p)** and as a zoomed in view of the same image plane in **(b)**, **(e)**, **(h)**, **(k)**, **(n)**, and **(q)**. The solid DVH curves in **(c)**, **(f)**, **(i)**, **(l)**, **(o)**, and **(r)** indicate the nominal values and the shaded regions the range of DVH values during the two evaluations, A and B. A line indicating a 90% probability, $p_{90\%}$, of being to the right of that curve is drawn for both evaluations for the clinical target volume (CTV). An analogous line indicating a 90% probability of protecting an organ of interest (OOI) against higher doses is indicated for only the evaluations performed with the evaluation image set A.

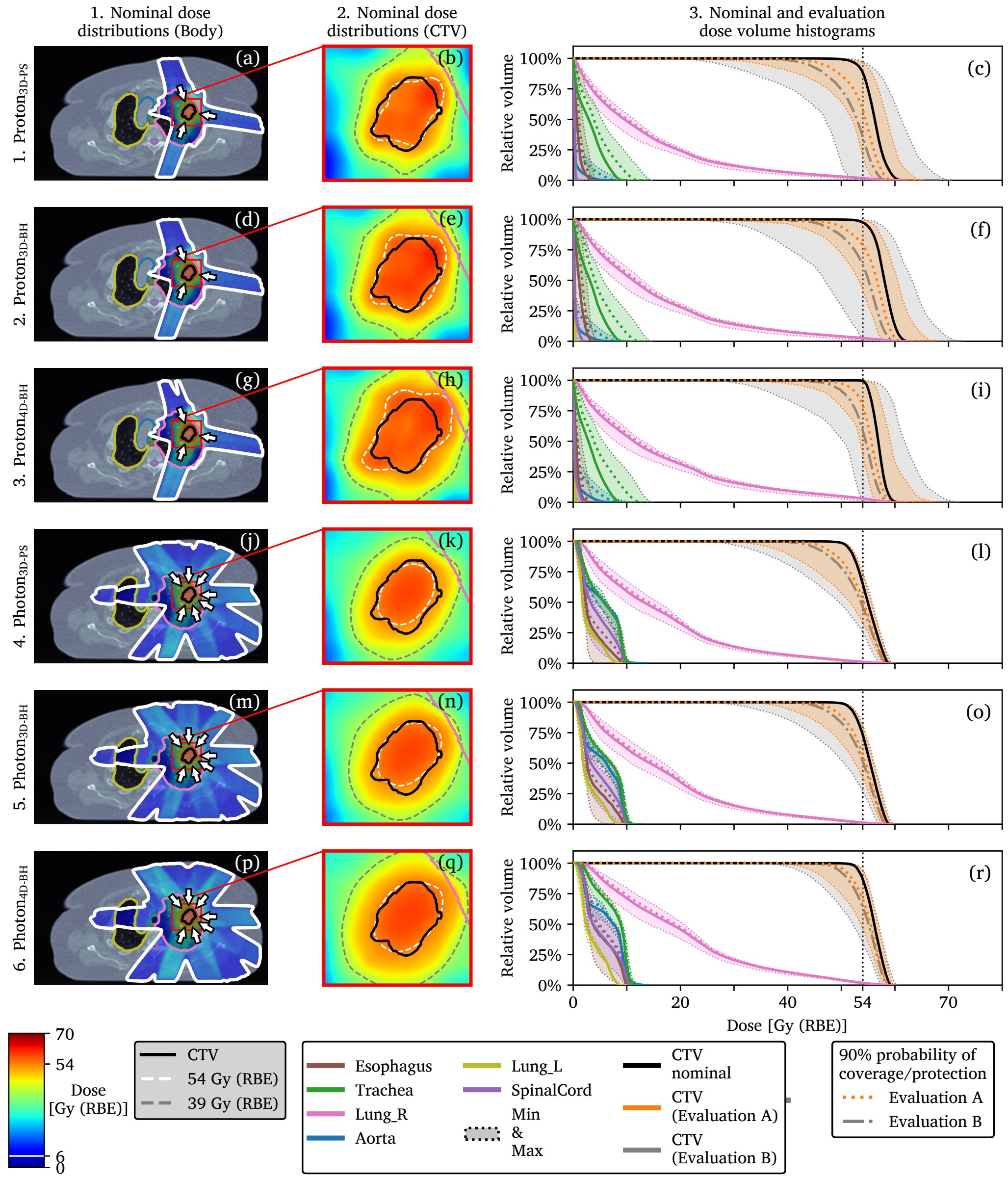

Supplementary Figure S3: Dose distributions and dose volume histograms (DVH) shown per plan for patient 3 with CTV size 7.8 cm^3 and tumor location above the carina in the right lung. The nominal dose distributions are shown for the entire transversal image plane that intersects the center of the tumor in **(a)**, **(d)**, **(g)**, **(j)**, **(m)**, and **(p)** and as a zoomed in view of the same image plane in **(b)**, **(e)**, **(h)**, **(k)**, **(n)**, and **(q)**. The solid DVH curves in **(c)**, **(f)**, **(i)**, **(l)**, **(o)**, and **(r)** indicate the nominal values and the shaded regions the range of DVH values during the two evaluations, A and B. A line indicating a 90% probability, $p_{90\%}$, of being to the right of that curve is drawn for both evaluations for the clinical target volume (CTV). An analogous line indicating a 90% probability of protecting an organ of interest (OOI) against higher doses is indicated for only the evaluations performed with the evaluation image set A.

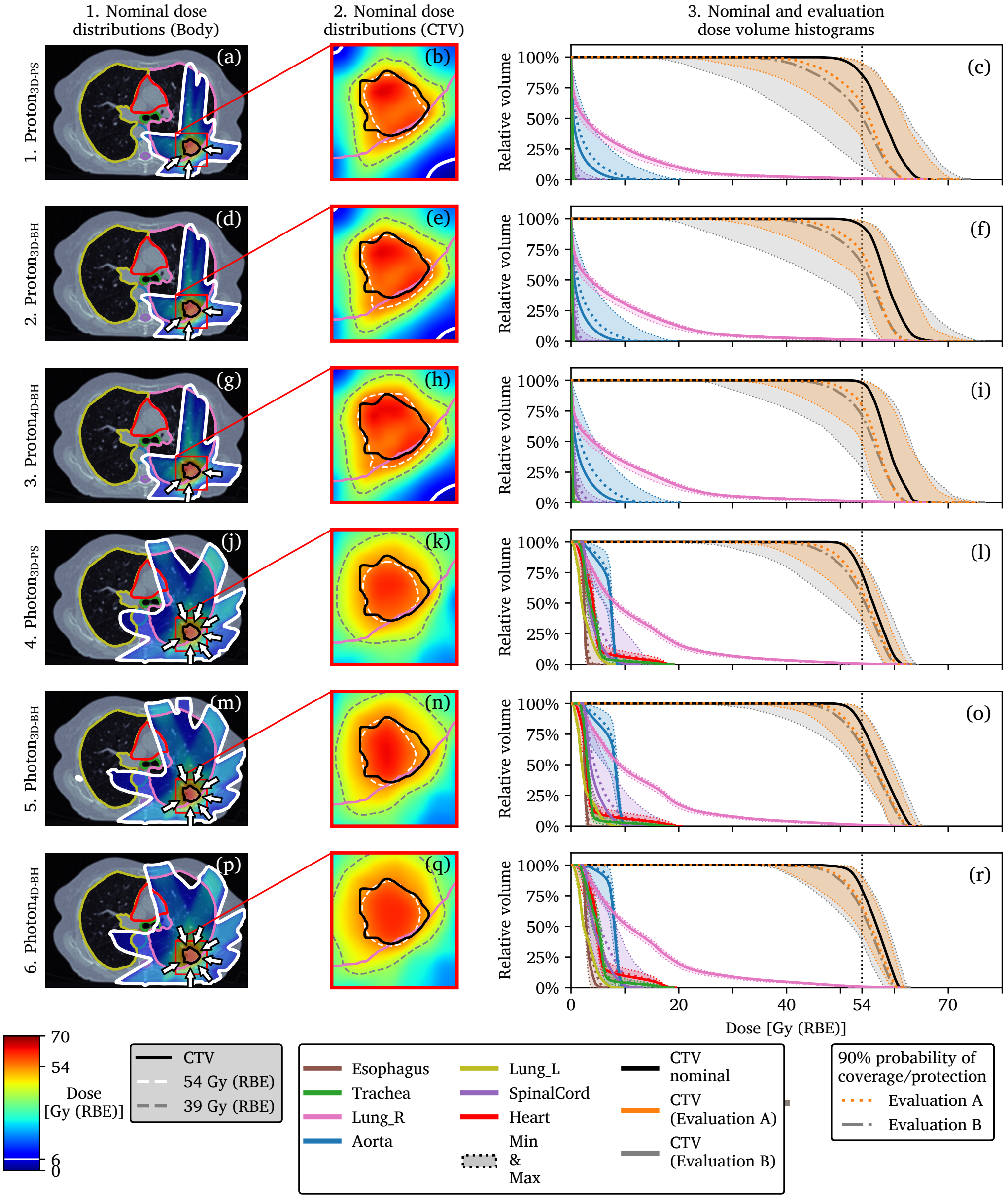

Supplementary Figure S4: Dose distributions and dose volume histograms (DVH) shown per plan for patient 4 with CTV size 7.5 cm^3 and tumor location at the level of the carina in the right lung. The nominal dose distributions are shown for the entire transversal image plane that intersects the center of the tumor in **(a)**, **(d)**, **(g)**, **(j)**, **(m)**, and **(p)** and as a zoomed in view of the same image plane in **(b)**, **(e)**, **(h)**, **(k)**, **(n)**, and **(q)**. The solid DVH curves in **(c)**, **(f)**, **(i)**, **(l)**, **(o)**, and **(r)** indicate the nominal values and the shaded regions the range of DVH values during the two evaluations, A and B. A line indicating a 90% probability, $p_{90\%}$, of being to the right of that curve is drawn for both evaluations for the clinical target volume (CTV). An analogous line indicating a 90% probability of protecting an organ of interest (OOI) against higher doses is indicated for only the evaluations performed with the evaluation image set A.

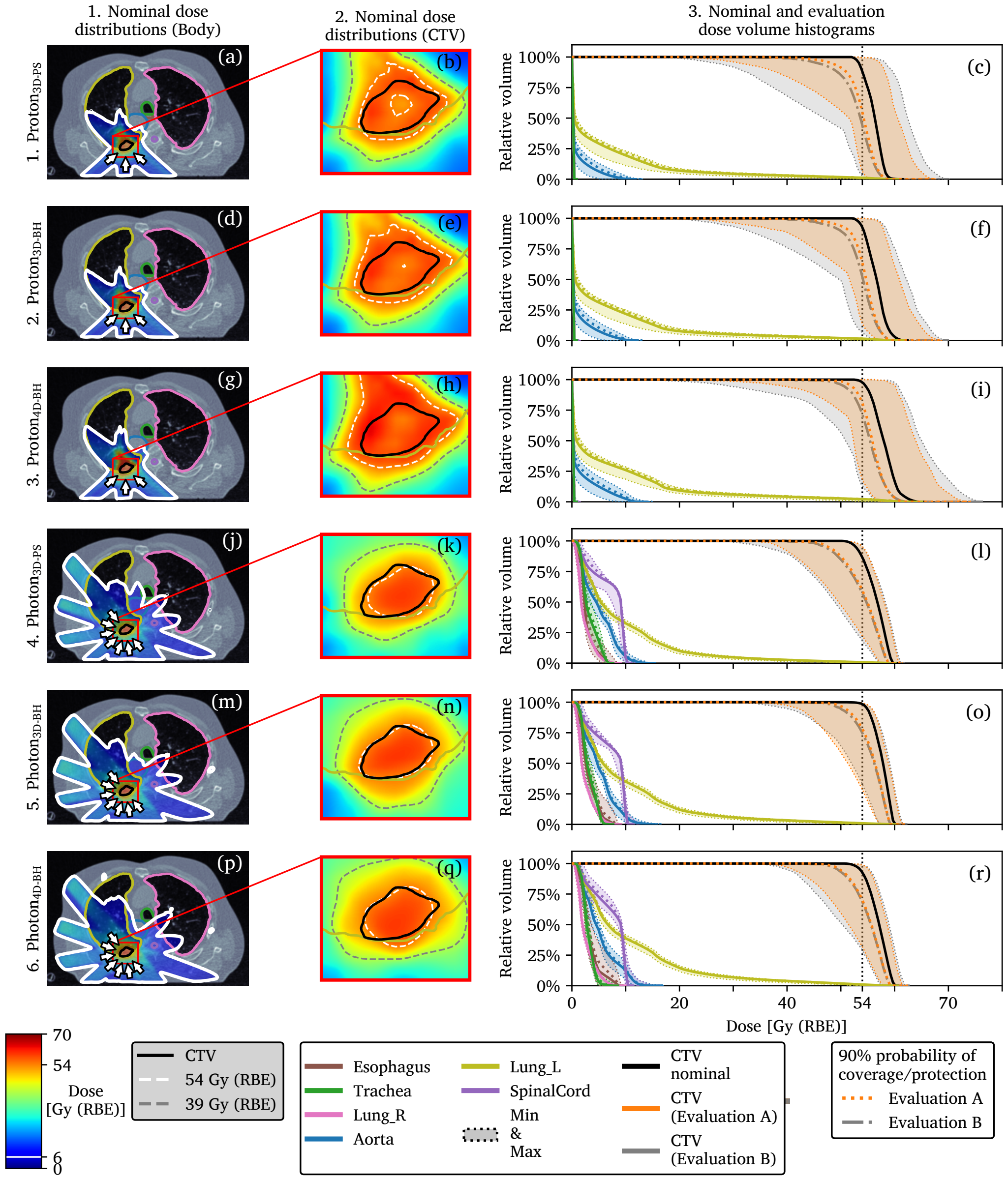

Supplementary Figure S5: Dose distributions and dose volume histograms (DVH) shown per plan for patient 5 with CTV size 4.1 cm^3 and tumor location at the level of the carina in the left lung. The nominal dose distributions are shown for the entire transversal image plane that intersects the center of the tumor in **(a)**, **(d)**, **(g)**, **(j)**, **(m)**, and **(p)** and as a zoomed in view of the same image plane in **(b)**, **(e)**, **(h)**, **(k)**, **(n)**, and **(q)**. The solid DVH curves in **(c)**, **(f)**, **(i)**, **(l)**, **(o)**, and **(r)** indicate the nominal values and the shaded regions the range of DVH values during the two evaluations, A and B. A line indicating a 90% probability, $p_{90\%}$, of being to the right of that curve is drawn for both evaluations for the clinical target volume (CTV). An analogous line indicating a 90% probability of protecting an organ of interest (OOI) against higher doses is indicated for only the evaluations performed with the evaluation image set A.

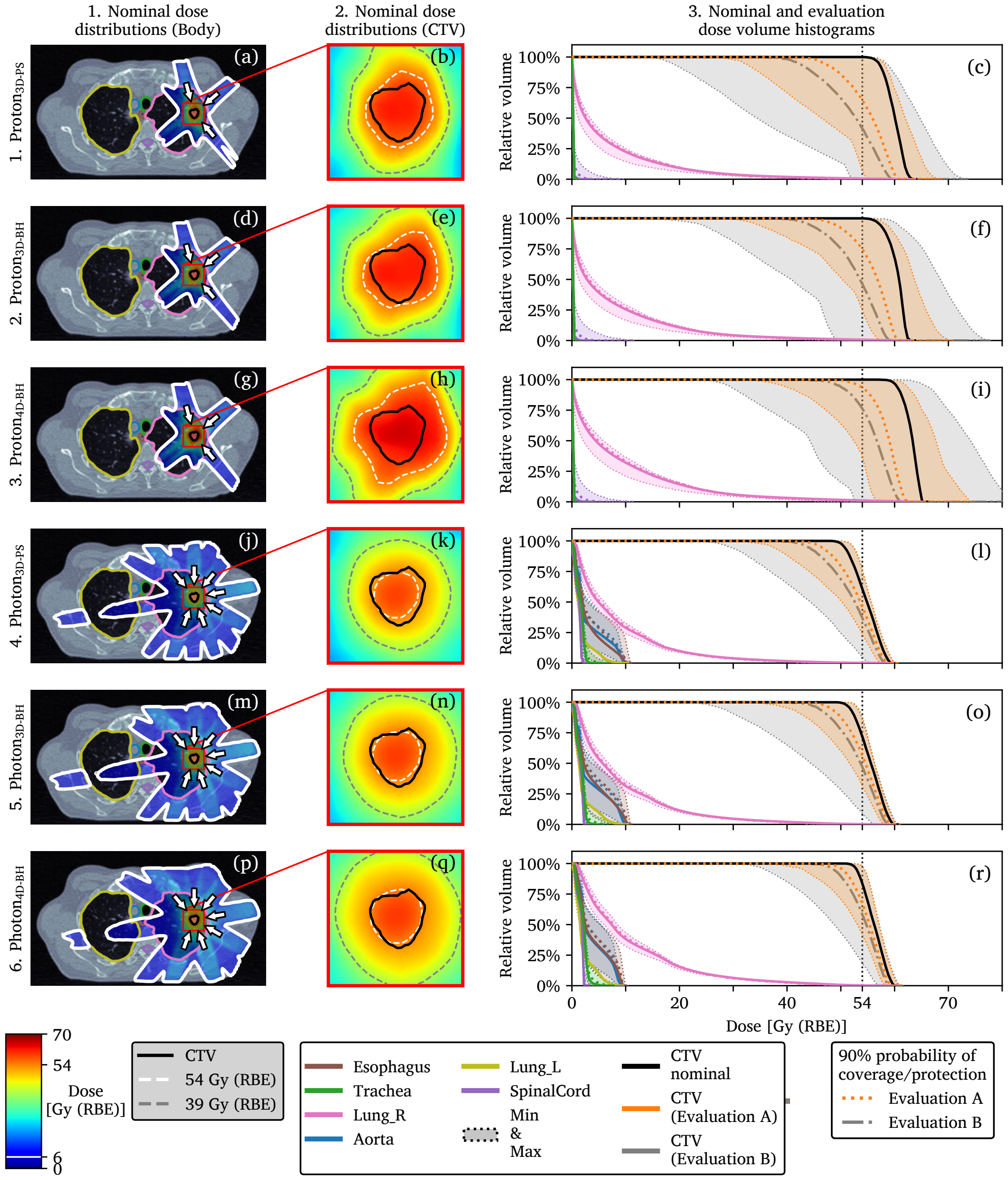

Supplementary Figure S6: Dose distributions and dose volume histograms (DVH) shown per plan for patient 6 with CTV size 1.4 cm^3 and tumor location above the carina in the right lung. The nominal dose distributions are shown for the entire transversal image plane that intersects the center of the tumor in **(a)**, **(d)**, **(g)**, **(j)**, **(m)**, and **(p)** and as a zoomed in view of the same image plane in **(b)**, **(e)**, **(h)**, **(k)**, **(n)**, and **(q)**. The solid DVH curves in **(c)**, **(f)**, **(i)**, **(l)**, **(o)**, and **(r)** indicate the nominal values and the shaded regions the range of DVH values during the two evaluations, A and B. A line indicating a 90% probability, $p_{90\%}$, of being to the right of that curve is drawn for both evaluations for the clinical target volume (CTV). An analogous line indicating a 90% probability of protecting an organ of interest (OOI) against higher doses is indicated for only the evaluations performed with the evaluation image set A.

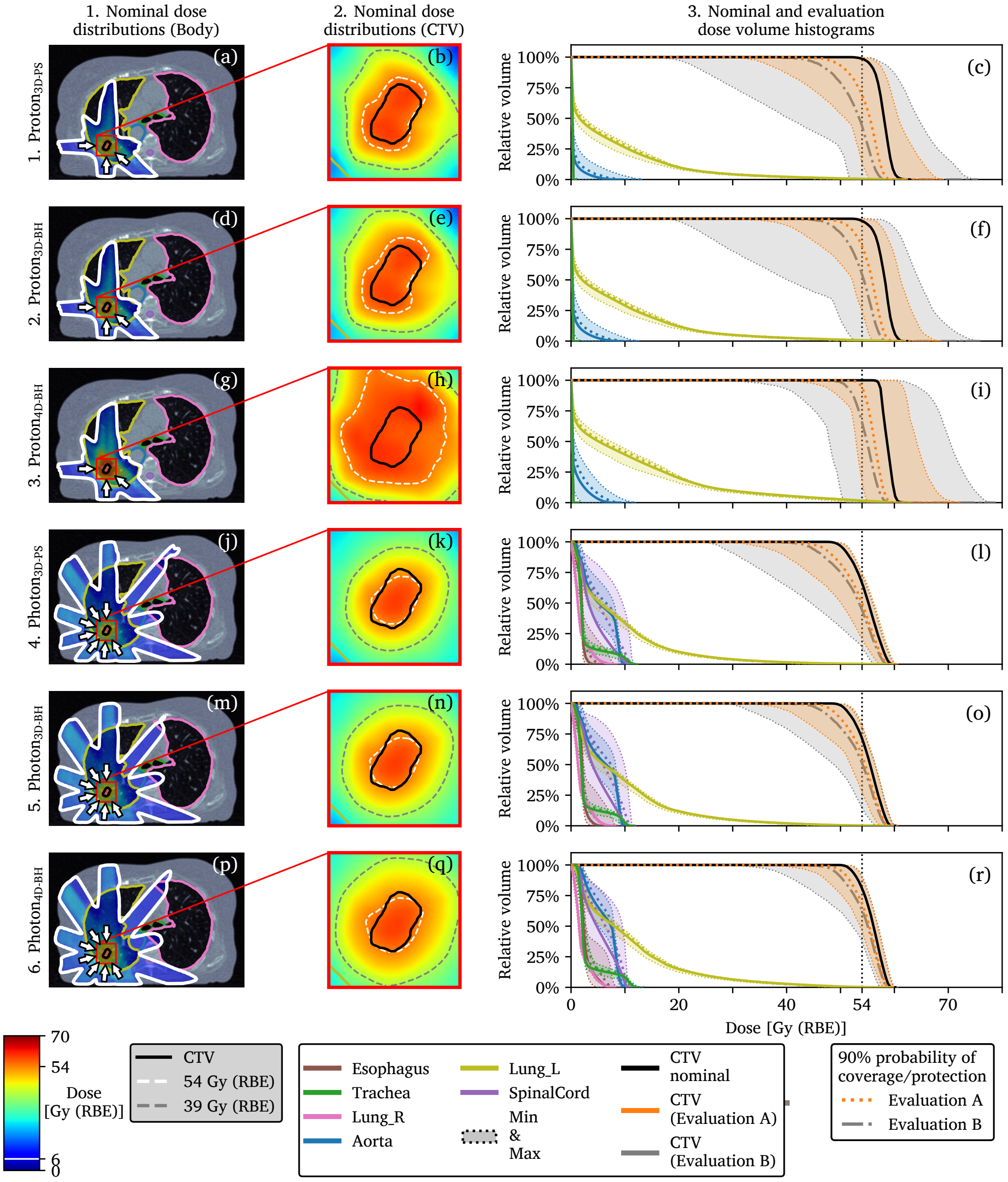

Supplementary Figure S7: Dose distributions and dose volume histograms (DVH) shown per plan for patient 7 with CTV size 1.7 cm^3 and tumor location above the carina in the left lung. The nominal dose distributions are shown for the entire transversal image plane that intersects the center of the tumor in **(a)**, **(d)**, **(g)**, **(j)**, **(m)**, and **(p)** and as a zoomed in view of the same image plane in **(b)**, **(e)**, **(h)**, **(k)**, **(n)**, and **(q)**. The solid DVH curves in **(c)**, **(f)**, **(i)**, **(l)**, **(o)**, and **(r)** indicate the nominal values and the shaded regions the range of DVH values during the two evaluations, A and B. A line indicating a 90% probability, $p_{90\%}$, of being to the right of that curve is drawn for both evaluations for the clinical target volume (CTV). An analogous line indicating a 90% probability of protecting an organ of interest (OOI) against higher doses is indicated for only the evaluations performed with the evaluation image set A.

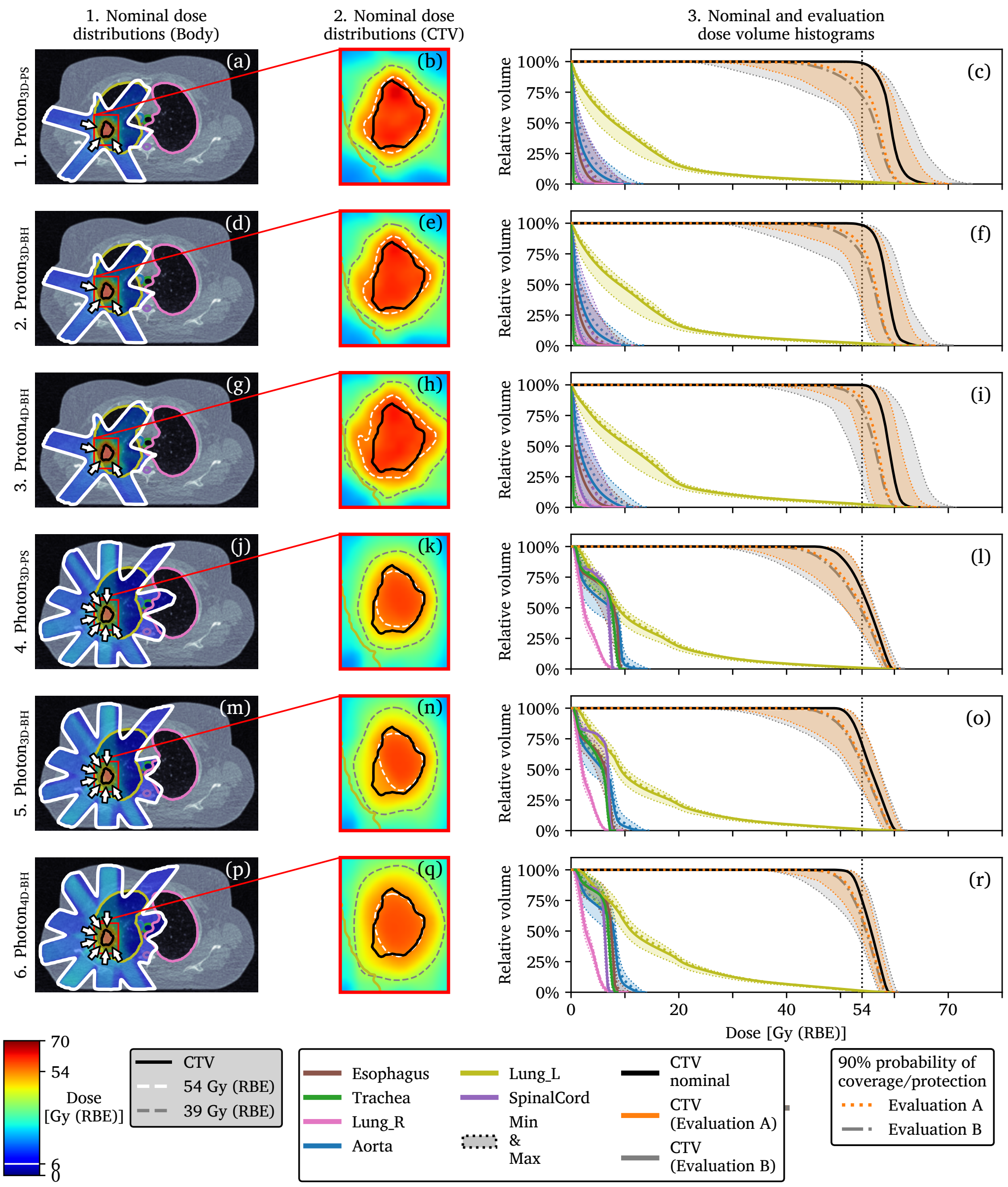

Supplementary Figure S8: Dose distributions and dose volume histograms (DVH) shown per plan for patient 8 with CTV size 10.1 cm^3 and tumor location above the carina in the left lung. The nominal dose distributions are shown for the entire transversal image plane that intersects the center of the tumor in **(a)**, **(d)**, **(g)**, **(j)**, **(m)**, and **(p)** and as a zoomed in view of the same image plane in **(b)**, **(e)**, **(h)**, **(k)**, **(n)**, and **(q)**. The solid DVH curves in **(c)**, **(f)**, **(i)**, **(l)**, **(o)**, and **(r)** indicate the nominal values and the shaded regions the range of DVH values during the two evaluations, A and B. A line indicating a 90% probability, $p_{90\%}$, of being to the right of that curve is drawn for both evaluations for the clinical target volume (CTV). An analogous line indicating a 90% probability of protecting an organ of interest (OOI) against higher doses is indicated for only the evaluations performed with the evaluation image set A.

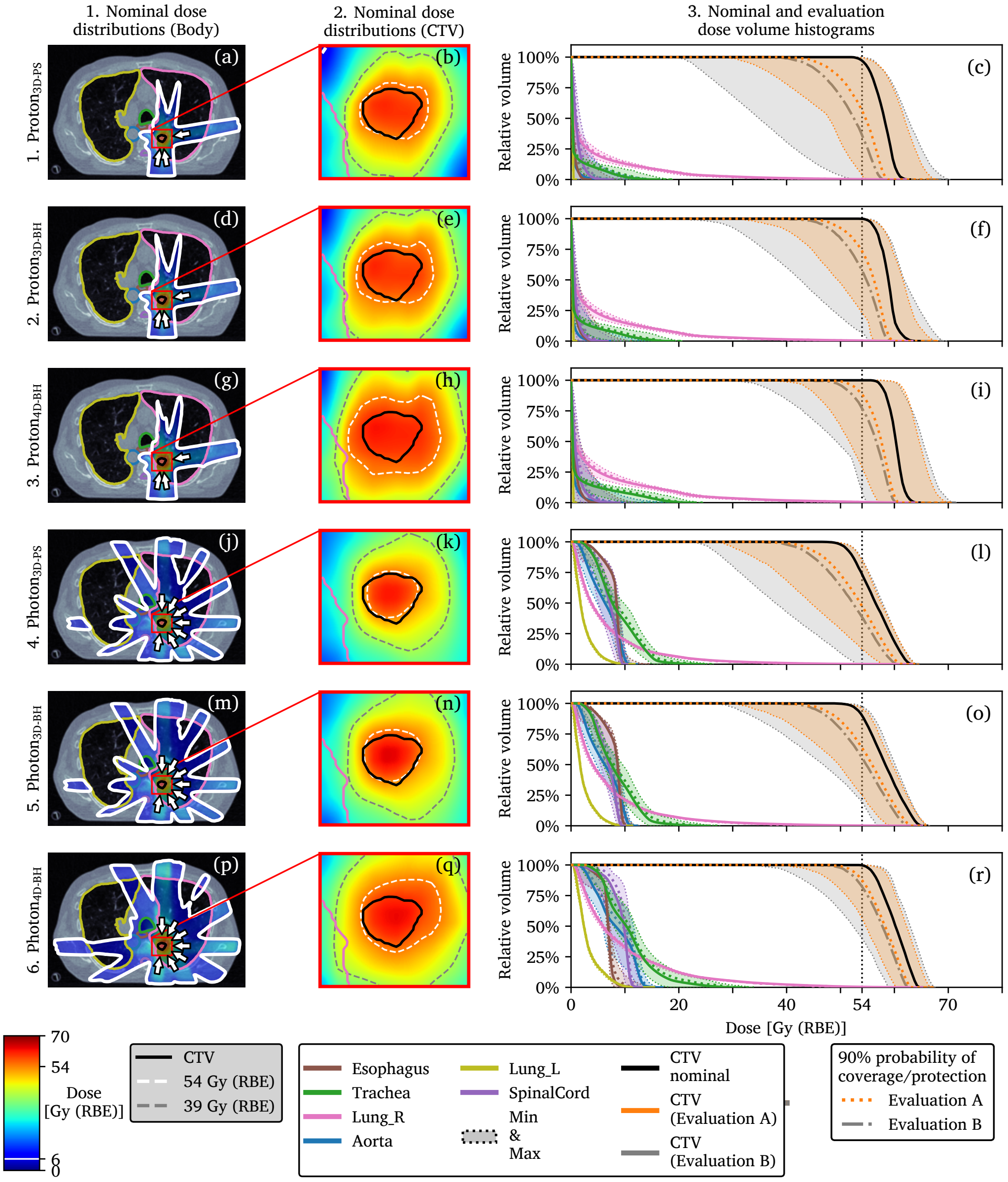

Supplementary Figure S9: Dose distributions and dose volume histograms (DVH) shown per plan for patient 9 with CTV size 1.3 cm^3 and tumor location below the carina in the right lung. The nominal dose distributions are shown for the entire transversal image plane that intersects the center of the tumor in **(a)**, **(d)**, **(g)**, **(j)**, **(m)**, and **(p)** and as a zoomed in view of the same image plane in **(b)**, **(e)**, **(h)**, **(k)**, **(n)**, and **(q)**. The solid DVH curves in **(c)**, **(f)**, **(i)**, **(l)**, **(o)**, and **(r)** indicate the nominal values and the shaded regions the range of DVH values during the two evaluations, A and B. A line indicating a 90% probability, $p_{90\%}$, of being to the right of that curve is drawn for both evaluations for the clinical target volume (CTV). An analogous line indicating a 90% probability of protecting an organ of interest (OOI) against higher doses is indicated for only the evaluations performed with the evaluation image set A.

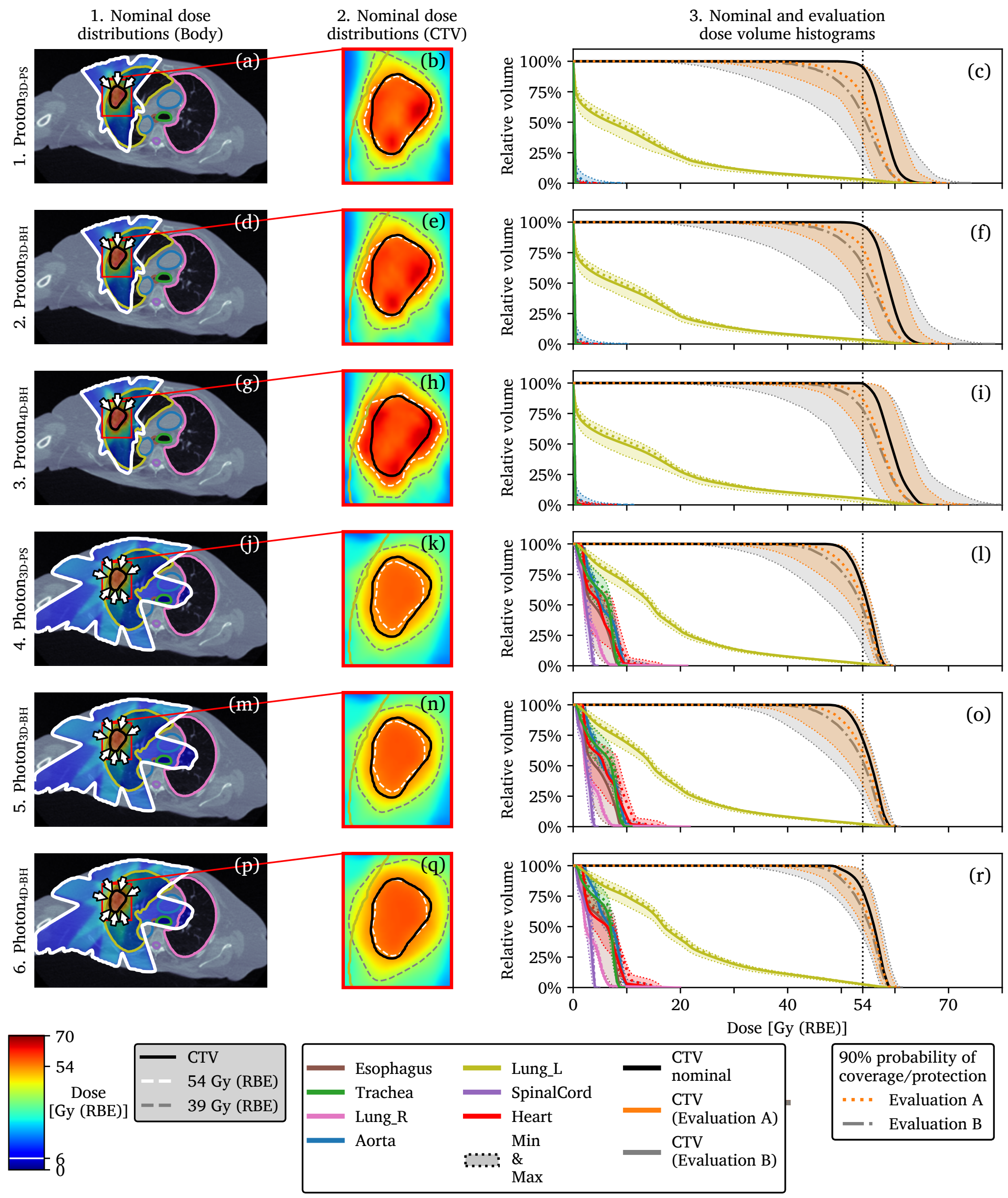

Supplementary Figure S10: Dose distributions and dose volume histograms (DVH) shown per plan for patient 10 with CTV size 21.5 cm^3 and tumor location at the level of the carina in the left lung. The nominal dose distributions are shown for the entire transversal image plane that intersects the center of the tumor in **(a)**, **(d)**, **(g)**, **(j)**, **(m)**, and **(p)** and as a zoomed in view of the same image plane in **(b)**, **(e)**, **(h)**, **(k)**, **(n)**, and **(q)**. The solid DVH curves in **(c)**, **(f)**, **(i)**, **(l)**, **(o)**, and **(r)** indicate the nominal values and the shaded regions the range of DVH values during the two evaluations, A and B. A line indicating a 90% probability, $p_{90\%}$, of being to the right of that curve is drawn for both evaluations for the clinical target volume (CTV). An analogous line indicating a 90% probability of protecting an organ of interest (OOI) against higher doses is indicated for only the evaluations performed with the evaluation image set A.

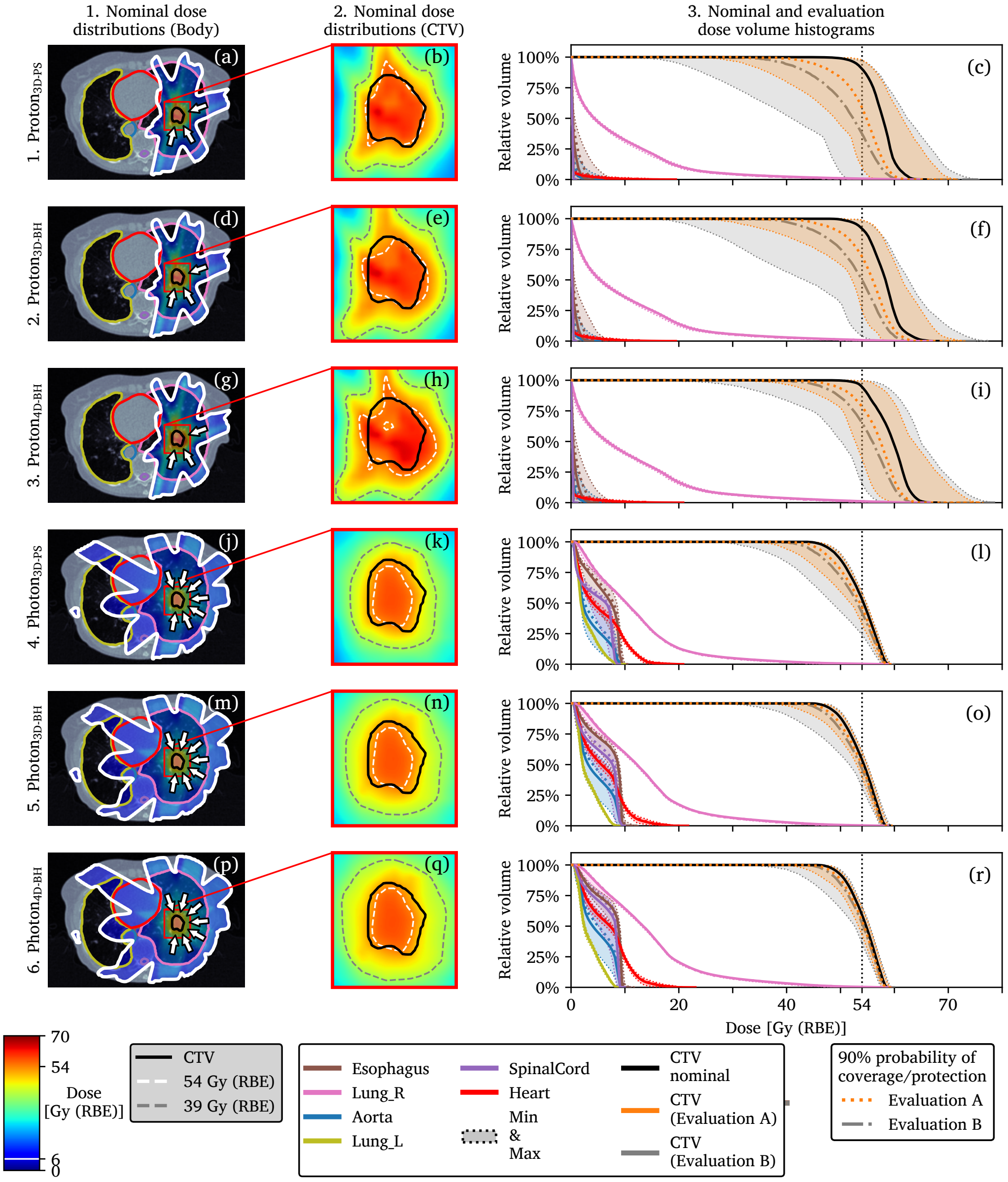

Supplementary Figure S11: Dose distributions and dose volume histograms (DVH) shown per plan for patient 11 with CTV size 4.8 cm^3 and tumor location below the carina in the right lung. The nominal dose distributions are shown for the entire transversal image plane that intersects the center of the tumor in **(a)**, **(d)**, **(g)**, **(j)**, **(m)**, and **(p)** and as a zoomed in view of the same image plane in **(b)**, **(e)**, **(h)**, **(k)**, **(n)**, and **(q)**. The solid DVH curves in **(c)**, **(f)**, **(i)**, **(l)**, **(o)**, and **(r)** indicate the nominal values and the shaded regions the range of DVH values during the two evaluations, A and B. A line indicating a 90% probability, $p_{90\%}$, of being to the right of that curve is drawn for both evaluations for the clinical target volume (CTV). An analogous line indicating a 90% probability of protecting an organ of interest (OOI) against higher doses is indicated for only the evaluations performed with the evaluation image set A.

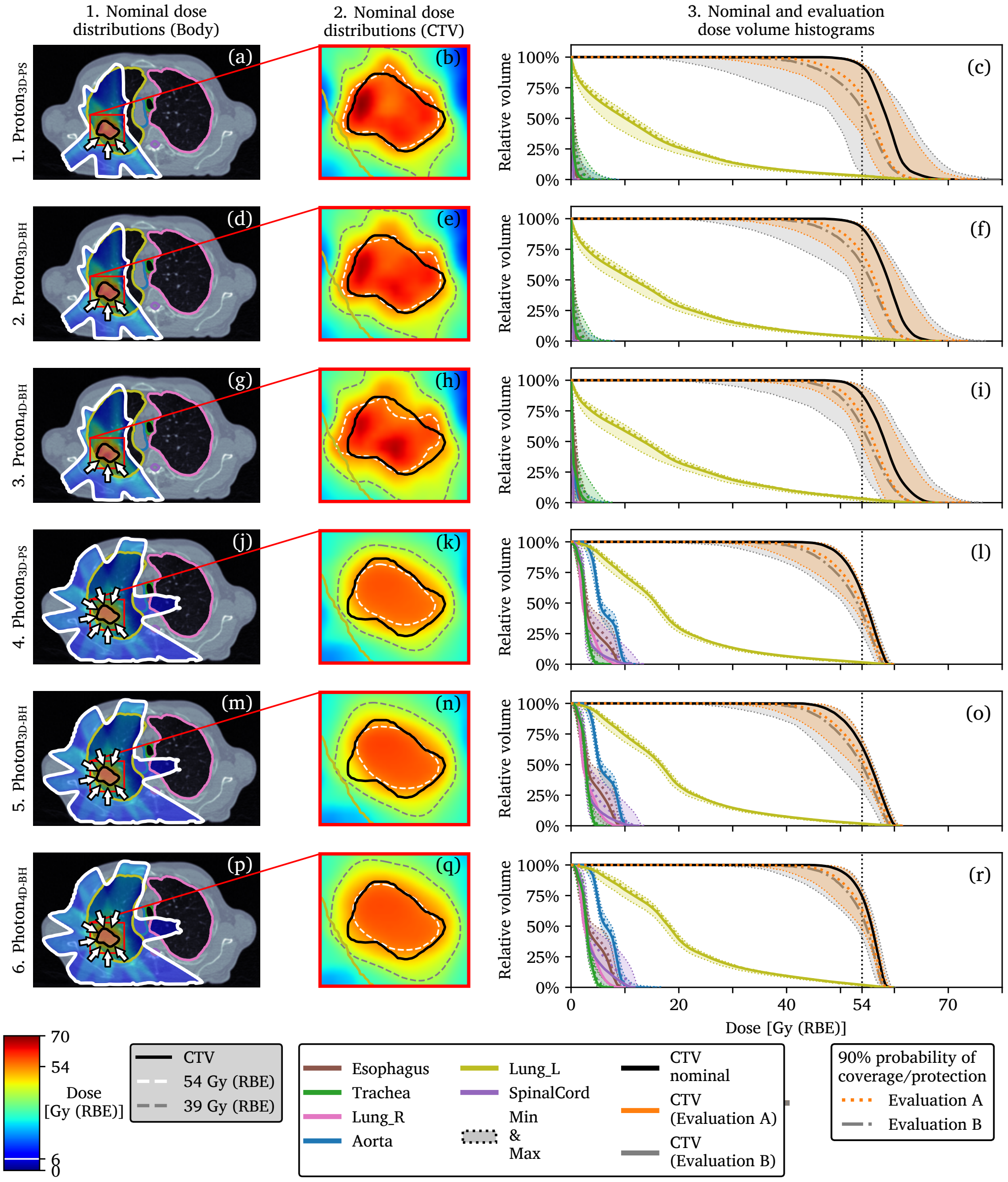

Supplementary Figure S12: Dose distributions and dose volume histograms (DVH) shown per plan for patient 12 with CTV size 19.2 cm^3 and tumor location above the carina in the left lung. The nominal dose distributions are shown for the entire transversal image plane that intersects the center of the tumor in **(a)**, **(d)**, **(g)**, **(j)**, **(m)**, and **(p)** and as a zoomed in view of the same image plane in **(b)**, **(e)**, **(h)**, **(k)**, **(n)**, and **(q)**. The solid DVH curves in **(c)**, **(f)**, **(i)**, **(l)**, **(o)**, and **(r)** indicate the nominal values and the shaded regions the range of DVH values during the two evaluations, A and B. A line indicating a 90% probability, $p_{90\%}$, of being to the right of that curve is drawn for both evaluations for the clinical target volume (CTV). An analogous line indicating a 90% probability of protecting an organ of interest (OOI) against higher doses is indicated for only the evaluations performed with the evaluation image set A.

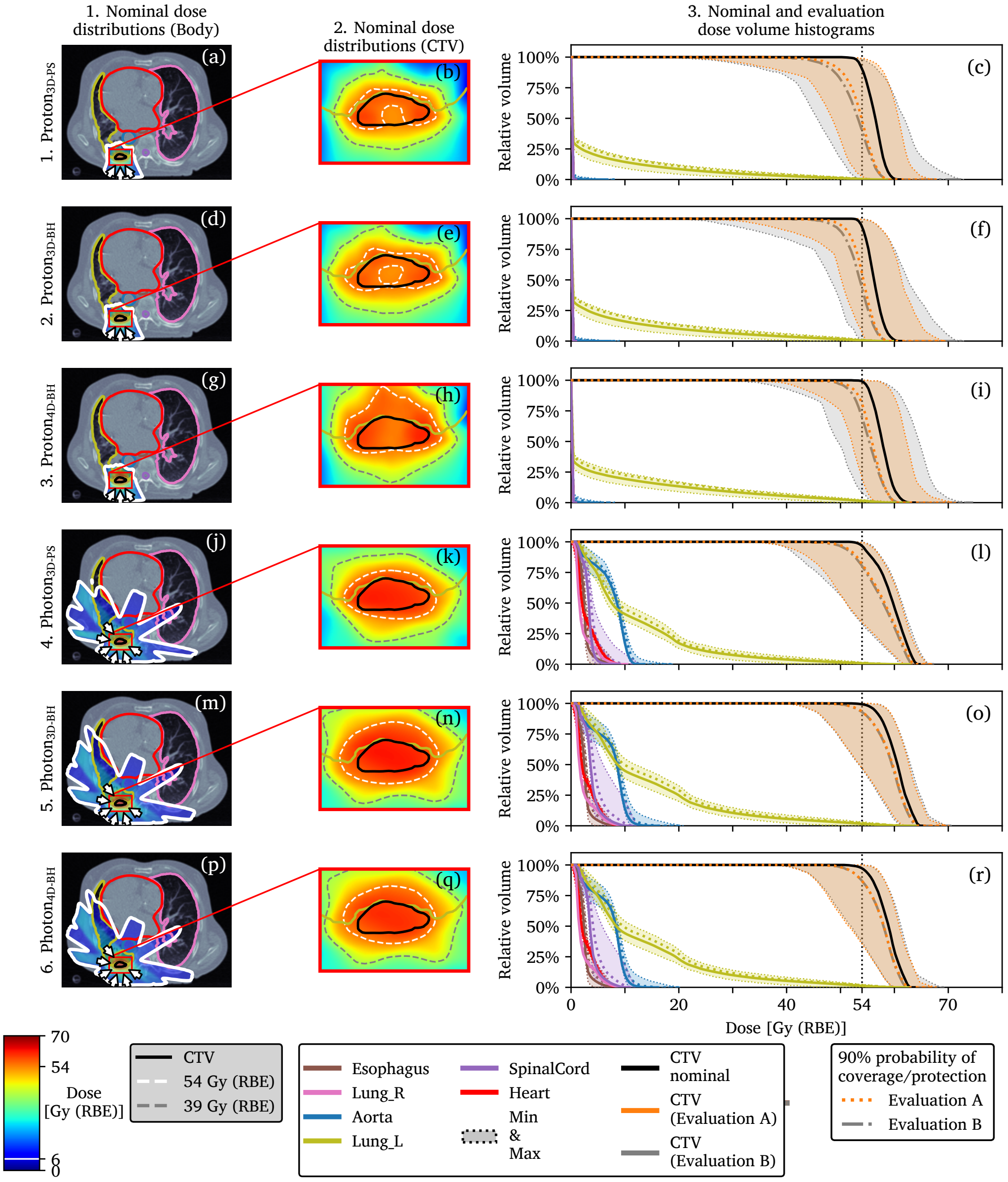

Supplementary Figure S13: Dose distributions and dose volume histograms (DVH) shown per plan for patient 13 with CTV size 5.5 cm^3 and tumor location below the carina in the left lung. The nominal dose distributions are shown for the entire transversal image plane that intersects the center of the tumor in **(a)**, **(d)**, **(g)**, **(j)**, **(m)**, and **(p)** and as a zoomed in view of the same image plane in **(b)**, **(e)**, **(h)**, **(k)**, **(n)**, and **(q)**. The solid DVH curves in **(c)**, **(f)**, **(i)**, **(l)**, **(o)**, and **(r)** indicate the nominal values and the shaded regions the range of DVH values during the two evaluations, A and B. A line indicating a 90% probability, $p_{90\%}$, of being to the right of that curve is drawn for both evaluations for the clinical target volume (CTV). An analogous line indicating a 90% probability of protecting an organ of interest (OOI) against higher doses is indicated for only the evaluations performed with the evaluation image set A.

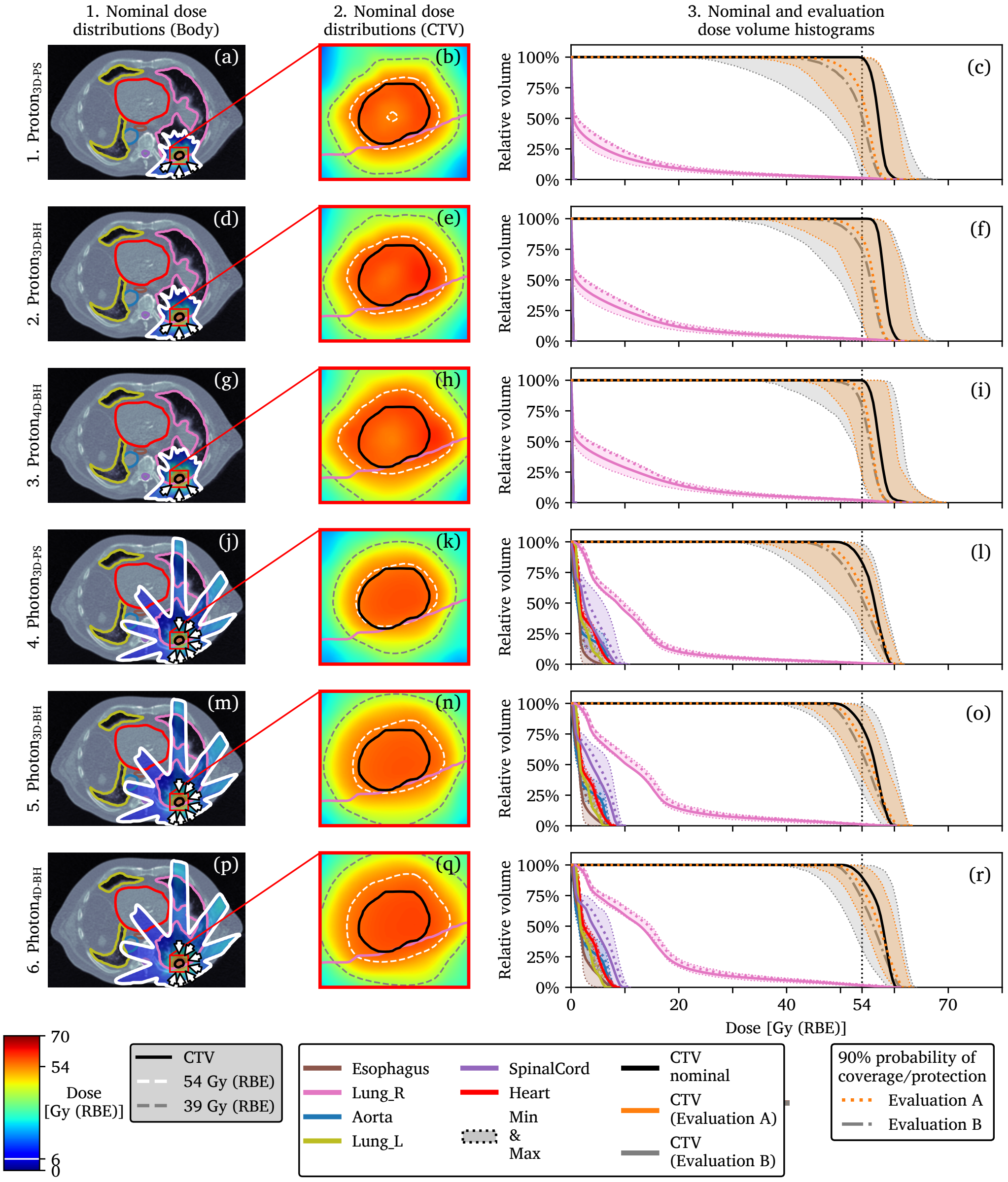

Supplementary Figure S14: Dose distributions and dose volume histograms (DVH) shown per plan for patient 14 with CTV size 3.9 cm^3 and tumor location below the carina in the right lung. The nominal dose distributions are shown for the entire transversal image plane that intersects the center of the tumor in **(a)**, **(d)**, **(g)**, **(j)**, **(m)**, and **(p)** and as a zoomed in view of the same image plane in **(b)**, **(e)**, **(h)**, **(k)**, **(n)**, and **(q)**. The solid DVH curves in **(c)**, **(f)**, **(i)**, **(l)**, **(o)**, and **(r)** indicate the nominal values and the shaded regions the range of DVH values during the two evaluations, A and B. A line indicating a 90% probability, $p_{90\%}$, of being to the right of that curve is drawn for both evaluations for the clinical target volume (CTV). An analogous line indicating a 90% probability of protecting an organ of interest (OOI) against higher doses is indicated for only the evaluations performed with the evaluation image set A.

3 Summarized CTV $D_{50\%}$ per plan for all patients

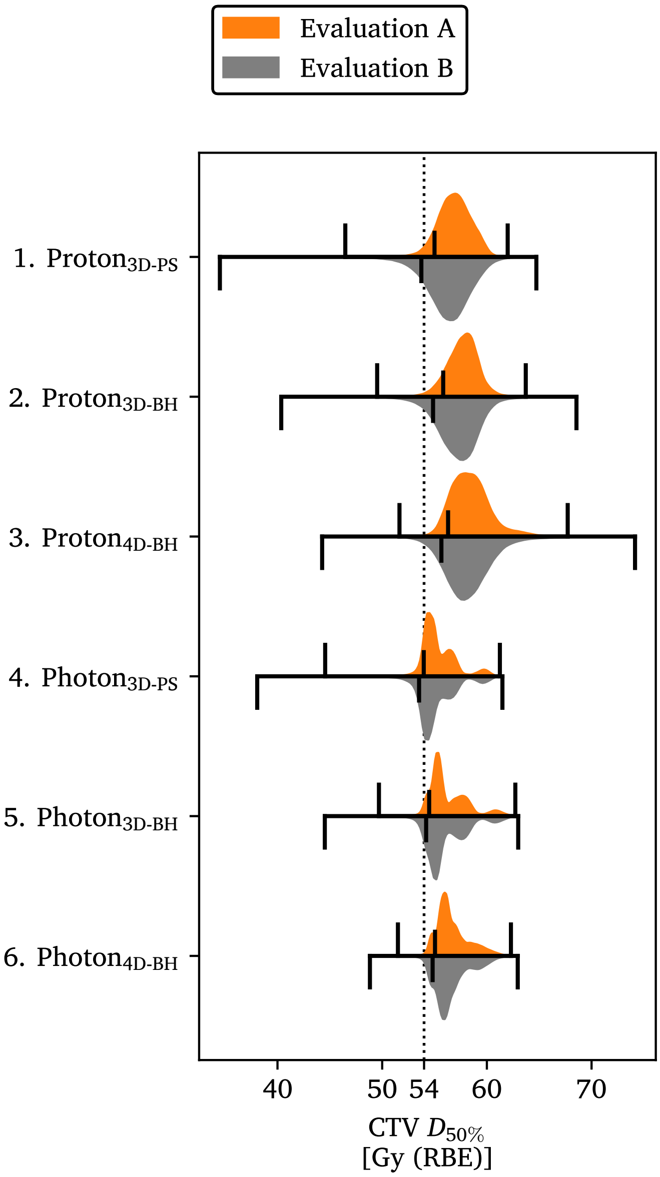

Supplementary Figure S15: Resulting distributions of CTV $D_{50\%}$ for each plan summarized for all 14 patients for both sets of evaluation images. The results from the two evaluation image sets are shown as orange and gray violin plots. Their $p_{90\%}$ value is indicated by the shorter black bar on the interval. It can be seen that the only plans that had an $p_{90\%}$ CTV $D_{50\%}$ that was below 54 Gy was the two 3D plans, $\text{proton}_{\text{3D-PS}}$ and $\text{photon}_{\text{3D-PS}}$, but only for the image set deformed with larger variations in breath-hold tumor position reproducibility shown in gray.

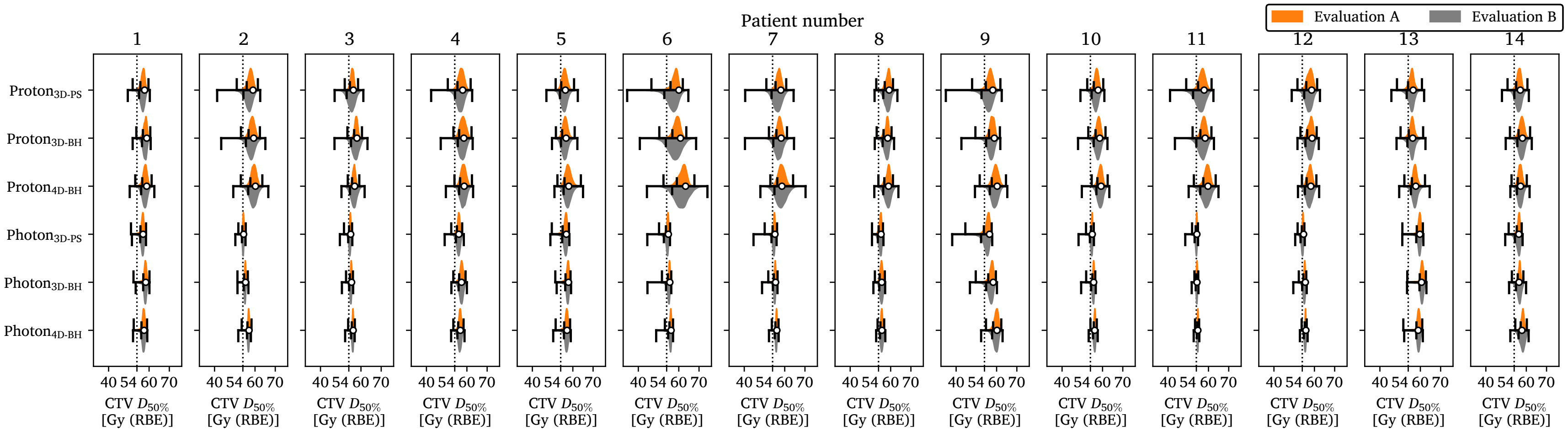

Supplementary Figure S16: Resulting dose covering 50% ($D_{50\%}$) of the clinical target volume (CTV) plotted for each treatment plan and patient. The results from the two evaluation image sets, A and B, are shown as orange and gray violin plots. Their $p_{90\%}$ value is indicated by the shorter black bar on the interval spanned by the smallest and largest values from the probabilistic evaluation, indicated by the longer black bars. The results from the evaluation performed with image set A is presented in the upper halves of the individual plots and the results from the evaluation performed with image set B in the lower. The nominal CTV $D_{50\%}$ is shown as a white dot.
